# Protein nanofibrils and their use as building blocks of sustainable materials

**DOI:** 10.1039/d1ra06878d

**Published:** 2021-12-08

**Authors:** Christofer Lendel, Niclas Solin

**Affiliations:** Department of Chemistry, KTH Royal Institute of Technology Teknikringen 30 SE-100 44 Stockholm Sweden lendel@kth.se; Department of Physics, Chemistry, and Biology, Electronic and Photonic Materials, Biomolecular and Organic Electronics, Linköping University Linköping 581 83 Sweden niclas.solin@liu.se

## Abstract

The development towards a sustainable society requires a radical change of many of the materials we currently use. Besides the replacement of plastics, derived from petrochemical sources, with renewable alternatives, we will also need functional materials for applications in areas ranging from green energy and environmental remediation to smart foods. Proteins could, with their intriguing ability of self-assembly into various forms, play important roles in all these fields. To achieve that, the code for how to assemble hierarchically ordered structures similar to the protein materials found in nature must be cracked. During the last decade it has been demonstrated that amyloid-like protein nanofibrils (PNFs) could be a steppingstone for this task. PNFs are formed by self-assembly in water from a range of proteins, including plant resources and industrial side streams. The nanofibrils display distinct functional features and can be further assembled into larger structures. PNFs thus provide a framework for creating ordered, functional structures from the atomic level up to the macroscale. This review address how industrial scale protein resources could be transformed into PNFs and further assembled into materials with specific mechanical and functional properties. We describe what is required from a protein to form PNFs and how the structural properties at different length scales determine the material properties. We also discuss potential chemical routes to modify the properties of the fibrils and to assemble them into macroscopic structures.

## Abstract 2

Protein nanofibrils produced from renewable resources provide opportunities to create novel materials for sustainable development.

## Graphical abstract

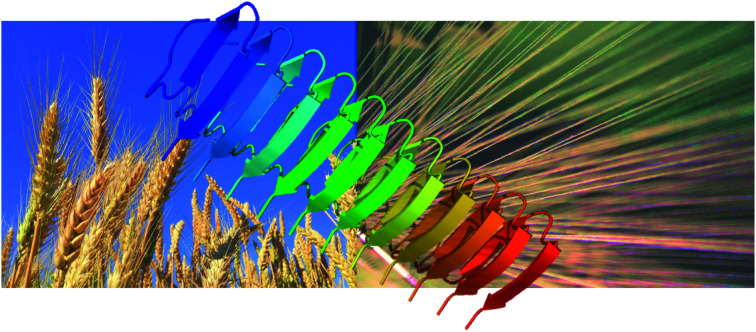

## 1. Introduction

Materials based on self-assembly are attractive as complex structures can be obtained from simple precursors employing simple procedures. Self-assembly processes of proteins are intimately linked with their biological function, and many high-performance materials in nature are made from proteins. A wide range of mechanical properties are observed for these materials, from the super-elastic, rubber-like resilin in insects^1^ to spider silk with higher toughness than both steel and Kevlar.^2^ Proteins also embody the scaffolds for many biocomposites, *e.g.* bone and seashells,^3^ and functional assemblies such as the photosynthetic systems where a protein scaffold helps to organize chromophores enabling harvesting of light. Natural protein materials are built from evolutionary optimized building blocks that are assembled with a hierarchical architecture at all structural levels, from the protein molecules to the macroscopic shape that we can see and touch. As individual molecules, proteins can thus be considered as a little “nanotechnological wonders” that rapidly folds into an ordered structure—with outstanding placement precision of individual atoms—optimised for a particular function through evolution. However, to achieve placement precision between molecules is more challenging. This is a considerable challenge for manmade materials that often have well-defined organization only at one structural level.^4^

The richness in chemical and physical properties of proteins††We will write ‘protein’ when we refer to naturally occurring molecules (even if they have been modified by synthetic biology). ‘Polypeptide’ is used as a more general term including all linear biopolymer chains build from amino acids. ‘Peptide’ refers to a short polypeptide. comes from their unique build-up. Proteins are polymers assembled from up to 20 different amino acid residues that, unlike other polymers, are combined in a specified order. The structure of proteins is described in terms of primary (amino acid sequence), secondary (local structural elements such as α-helices and β-strands), tertiary (the arrangement of the structural elements in three dimensions), and quaternary (arrangement of different molecular subunits) structures. In order to function (*e.g.* as a catalyst) most proteins adopt well-defined structure in three dimensions, referred to as the native state. As the native state is only one of many possible conformations, the ability to form a well-defined native state requires the presence of attractive intramolecular interactions to compensate for the low conformational entropy of the native state. As individual non-covalent interactions are weak, a highly optimized primary structure with the ability to create enough attractive contacts in the native state is required. Under conditions where the native state is destabilized – a process known as denaturation – the polymer chain can thus explore alternative conformations. The smallest known proteins with a stable native structure are built up from 10–20 amino acid residues.^5,6^ Cyclic chains can be as small as five amino acid residues.^7^ Notably, if intermolecular interactions are included even smaller peptides can form ordered and stable structures. In fact, peptides consisting of only two amino acid residues can form structures where intermolecular hydrogen bonds between β-strands generated extensive β-sheets.^8^ This can be viewed as a supramolecular polymeric structure, where the individual peptide molecules constitute monomers and the resulting array of β-sheets the supramolecular polymer.

**Fig. 1:**
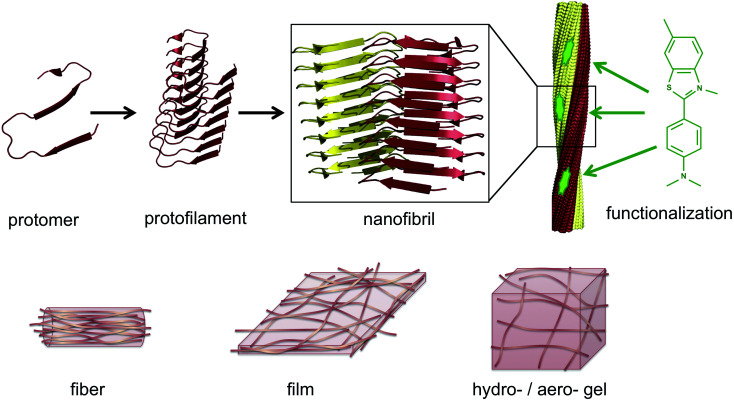
Illustration of the architecture of amyloid-like PNFs and the materials that can be assembled from them. A large number of protomers build up the protofilament, which is the smallest fibrillar structure. Two or more protofilaments then assemble into nanofibrils, *e.g.* as twisted ribbons. The nanofibrils display structural features that can be exploited for functionalization, here exemplified by a small organic dye (thioflavin T). On the lower row it is shown how PNFs can build up fibers, films or gels.

The same type of structures constitutes the basis for amyloid-like protein nanofibrils (PNFs). The fibril can be viewed as a supramolecular polymer formed by self-assembly, where individual protein molecules are the monomers. In the PNF the structural order within an individual protein molecule is translated into an ordered supramolecular structure that extends into dimensions of micrometres along one axis, whereas the dimensions of the perpendicular axis (*i.e.* the fibril diameter) are a few nanometres. The molecular building blocks are polypeptide chains oriented perpendicular to the fibril axis in β-sheet structures. Such an array of molecules in a ‘cross-β’ structure constitutes a protofilament. Several protofilaments can then be arranged in different ways on the nanoscale level to form the PNF (Fig. 1). The protein fibrils have an extremely large aspect ratio, giving them opportunities to form a range of different macroscopic materials/structures, including hydrogels, aerogels, structurally ordered films, and lyotropic liquid crystalline phases (Fig. 1). In addition, the fibril contains specific structural elements where external molecules may bind – a phenomena that opens for functionalization with non-protein components. And most important, the formation of β-sheet rich PNFs is now recognized as an inherent ability of the polypeptide main chain^9^ and PNFs could thus in principle be obtained from any protein.

Several excellent and comprehensive reviews, discussing amyloid from various perspectives, have recently been published, and the present review does not aim to cover all areas. The focus will be on PNFs formed from proteins that can be obtained in large quantities, preferably from plants or industrial waste-streams. However, as the research on amyloid formation from such sources is still in its infancy, some of the examples will be taken from other proteins. The aim is that these sections can act as inspiration for researchers aiming to further investigate materials science aspects of PNFs formed from plant proteins or proteins obtained from industrial waste-streams.

We will in the next section give a historical perspective on proteins and PNFs in material science with a specific focus on sustainability. In Section 3, the structural variations of protein fibrils will be discussed in more detail. Section 4 presents an overview of which proteins that can form PNFs and describes the mechanism of fibril formation in more detail. Functionalization of protein fibrils will be discussed in Section 5. Finally, the assembly into ordered micro–macroscale structures is described in Section 6, followed by a general outlook in Section 7.

## 2. Protein materials, protein nanofibrils, and sustainability

In a historical perspective, the soft protein materials employed by humans were obtained from natural renewable sources. Materials such as silk, wool, and leather, have been with us since times immemorial. These materials were developed by trial and error over time without access to current knowledge about protein molecules. The word protein itself is relatively recent and was coined in 1838 (in a private letter) by the Swedish scientist Jöns Jacob Berzelius. Instead, terms such as albuminous or gelatinous were employed to describe substances that we now know are made up of proteins. Sediments of this usage still remain in many languages. The latin word albumin is derived from *alba* meaning white, and in German *eiweiss-stoff* (*ei* = egg; *weiss* = white) was used to denote protein-like materials. In fact, this usage is still employed in east Asian languages: in Japanese the word *tanpaku* (
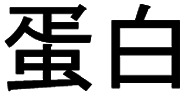
) combines the characters (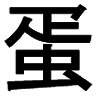 = egg) and (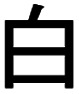 = white), and in modern Chinese the word *danbaizhi* (
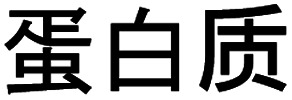
) combines the same two characters with (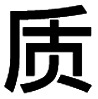); both *tanpaku* and *danbaizhi* are now used to denote proteins in general.

During the initial stage of the polymer industry (end of the 19^th^ century and the beginning 20^th^ century) all plastics were made from biopolymers, and most of them from renewable resources such as cellulose, shellac, and ebonite. Proteins were also intensively investigated but after the development of efficient methodologies for synthesis of organic polymers (based on raw materials from the petrochemical industry) in the mid 20^th^ century, the use of biopolymers went into steep decline. However, with the recent realization of the problematic aspects of non-biodegradable plastics and the realization that petrochemicals are a limited resource, there has been a dramatic increase of research about biopolymers in materials science. A large focus has been on carbohydrate- (*e.g.* cellulose and chitin) and DNA-based materials, but recently the investigation of proteins for applications as materials is also rising. From this perspective, it is very interesting that already in the early 20^th^ century engineers developed protein-based plastics from casein and soy protein.^10^ In fact, in the 1930s the automobile maker Ford extensively investigated soy protein as a material for cars.^11^

Proteins also have a unique niche in material science as edible materials. Besides their essential nutritional value, proteins and their structural arrangement also provide texture (*i.e.* determine the mechanical properties) in our foodstuff, *e.g.* the gluten network in bread, the muscle fibers (actin/myosin) in meat or melted cheese (casein) on a pizza, and are therefore vital for the taste experience. Hence, in our everyday lives we are producing and redesigning protein materials as we cook our meals. However, the close relation between proteins and food also highlight a critical question regarding proteins as a source of sustainable materials, as their use as materials will always compete with a potential use as animal feed or human food. A healthy diet requires intake of proteins, and throughout history a huge challenge for a large portion of humanity has been how to acquire protein-rich food. Production of meat from cattle demands huge resources, compared to *e.g.* growing plants,^12,13^ and throughout history meat has often been considered a luxury product in many regions of the world. With both an increasing population and increasing concerns regarding the sustainability of current food production the choice of food has a large impact on the global climate and environment as well as on our health.^14^ Taking a materials perspective on food it is easy to imagine how engineering of protein-based materials could also lead to novel and more sustainable foodstuff. For example, many western world consumers have a strong preference for animal-meat compared to meat replacement products. With the increased knowledge in self-assembly, it should be possible to produce artificial protein structures with attractive taste and texture. Here PNFs may play an important part as meat has a fibrous structure that may be mimicked through hierarchical assembly, while keeping in mind the potential safety issues of using engineered protein nanostructures.^15–18^

With the high environmental cost required for traditional production of meat there is currently a huge interest in converting protein waste streams into edible form – either for human food or as animal feed. In connection with these considerations, it is important to note that for applications not involving protein as a bulk material the amount of protein diverted from food supply will be small. For example, 1 litre of tightly packed protein material can in principle be employed as a 1 μm thick coating covering 1000 m^2^, or as a fibre of 1 μm in diameter having a total length of more than one billion meters. In other words, for applications involving thin films or microfibers a limited amount of protein materials can be employed to cover large areas. In addition, an economical value increase can be expected as one goes from applications as bulk materials, to functional materials, to components of active devices.^19^

Amyloid and PNFs have a long history originating in medicine but diverging into both food science and nanotechnology during the last 30–40 years. The name ‘amyloid’ was coined by Rudolf Virchow in 1854.^20^ Virchow found that a type of abnormal brain tissue could be stained by iodine. As it was known that starch may be stained by iodine Virchow assumed that the abnormal tissue was related to starch, and he used the name amyloid (from the latin *amylon* meaning starch and the suffix-*oid* used to indicate similarity) that had earlier been employed in botany for plant substance that could be stained by iodine. It was later found that the type of tissue investigated by Virchow contained large amount of nitrogen ruling out that it consisted of only carbohydrates; however, the name has stayed. Later it was found that certain dyes, *e.g.* Congo red, could be utilized to stain amyloid, and such methods still constitute one of the most common methods to detect the presence of amyloid. The name amyloid is thus strongly connected with diseases, and a range of different pathologies has been associated with formation of amyloid from different proteins.^9,21^ However, a large number of examples of functional amyloid has also been found, where the amyloid structure is beneficial for the organism^22^ showing that the structure *per se* is not pathogenic. Such functional amyloid has been found in variety of organisms, from mammals and insects to fungi and bacteria^23–25^ and recently also in plants.^26^ In addition, a range of proteins from plants, industrial waste streams or obtained as side-products during food production have been described to form amyloid-like structures *in vitro*. Generally the type of protein fibrils formed *in vivo* will be labelled as amyloid. If the same proteins are induced to form fibrils *in vitro* the material is typically labelled as amyloid-like. In the rest of this review, we will mainly employ the name protein nanofibril (PNF) when discussing protein structures formed *in vitro* from proteins not associated with pathogenic proteins. However, it should be noted that the different names correspond to one type of generic structure, and in certain places the name amyloid or amyloid-like may be employed. No toxic effects have so far been observed for these types of fibrils,^27–29^ which opens for large-scale production of PNFs from industrial or agricultural side streams.

**Fig. 2:**
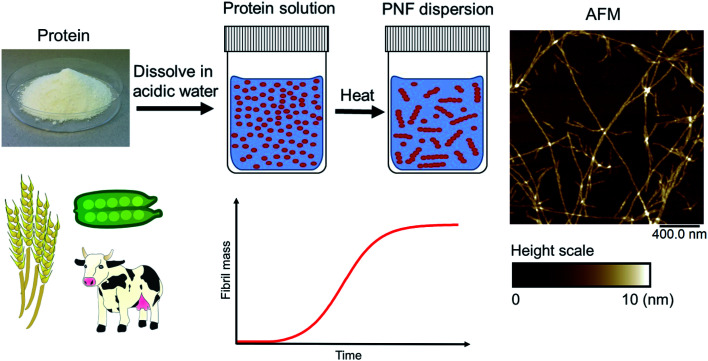
Schematic overview of a typical process for formation of PNFs. Whey protein is dissolved in acidic water (pH 2) and heated (80–90 °C), resulting in PNF formation. The process typically follows kinetics described by a sigmoidal curve. An illustrative AFM image of PNFs is shown.

Amyloid fibrils can be viewed as a protein fold requiring intermolecular interactions in contrast to the native fold that required strong intramolecular interactions. The practical ease by which such transformations can be performed in the lab is quite remarkable. In fact, the process was first described by Waugh in 1946 (ref. 30) even though the relation of the resulting materials with amyloid plaques were not known. When powder of bovine insulin is dissolved in weakly acidic water (giving a clear solution) and heated, the protein will aggregate resulting in a turbid sample. If analysed by atomic force microscopy (AFM) the turbid sample can be shown to contain fibrous objects (and sometimes also protein spherulites built up from such fibrous objects). The majority of fibrillation procedures are of similar operational simplicity, with common variations being for example addition of additives (*e.g.* salt or a co-solvent) or agitation of the reaction mixture during fibrillation. Fig. 2 shows an example of conversion of whey protein into PNFs – a process that will be described in more detail in Section 4. In spite of whey being a complex mixture of proteins and sugars the practical procedure is essentially the same as that described by Waugh in 1946. The protein is dissolved in acidic water and heated, whereupon PNFs are formed. The exact reaction conditions, including pH and temperature will depend on the specific protein, but in broad outline procedures of this type will typically be the first choice when investigating suitable fibrillation conditions for a given protein.

## 3. PNF structure and polymorphism

Design of hierarchical protein materials requires understanding of the structural properties at the different length scales. In this section we will describe the molecular structures and nanoscale features of PNFs while Section 6 deals with the assembly of fibrils into larger structures. The structural features of amyloid fibrils and materials made from such nanofibrils have been the topic for several reviews lately, see *e.g.* ref. 25 and 31–35. We will here give a brief overview and highlight selected aspects that we find interesting in relation to the scope of this review. Polymorphism, within the context of amyloid, is defined as different amyloid structures formed by the same polypeptide.^36^ There are three types of structural variations that give rise to polymorphism: the number of protofilaments in the amyloid fiber, the relative arrangement of the protofilaments and the conformation of the protomers in the protofilament.^36^ More than one type of variation can of course occur in the same fibril structure.

### 3.1 High resolution structures of PNFs

Structural biology of amyloid fibrils have developed rapidly during recent years, mainly thanks to technological development in magic angle spinning (MAS) solid-state nuclear magnetic resonance (NMR) spectroscopy^34^ and, in particular, single particle cryo electron microscopy (EM).^37,38^ Still, only a small portion of the protein structures in the Protein Data Bank (PDB) are amyloid-like fibrils and the majority of these are structures of fibrils formed from short peptide fragments (4–10 amino acid residues).^39^ This should be viewed in relation to the fact that “all” proteins could form amyloid (or even many different amyloid structures if polymorphism and fibrillation of hydrolysis products are considered). Moreover, human disease-related proteins (*e.g.* amyloid β related to Alzheimer's disease) are strongly over-represented among deposited structures but there are also a few functional amyloid structures (*e.g.* fungal protein Het-S^40^ and Drosophila Orb2 (ref. 41)). Notably, important model fibrils for material applications, *e.g.* from β-lactoglobulin or lysozyme, are still lacking high-resolution structure information.

**Fig. 3:**
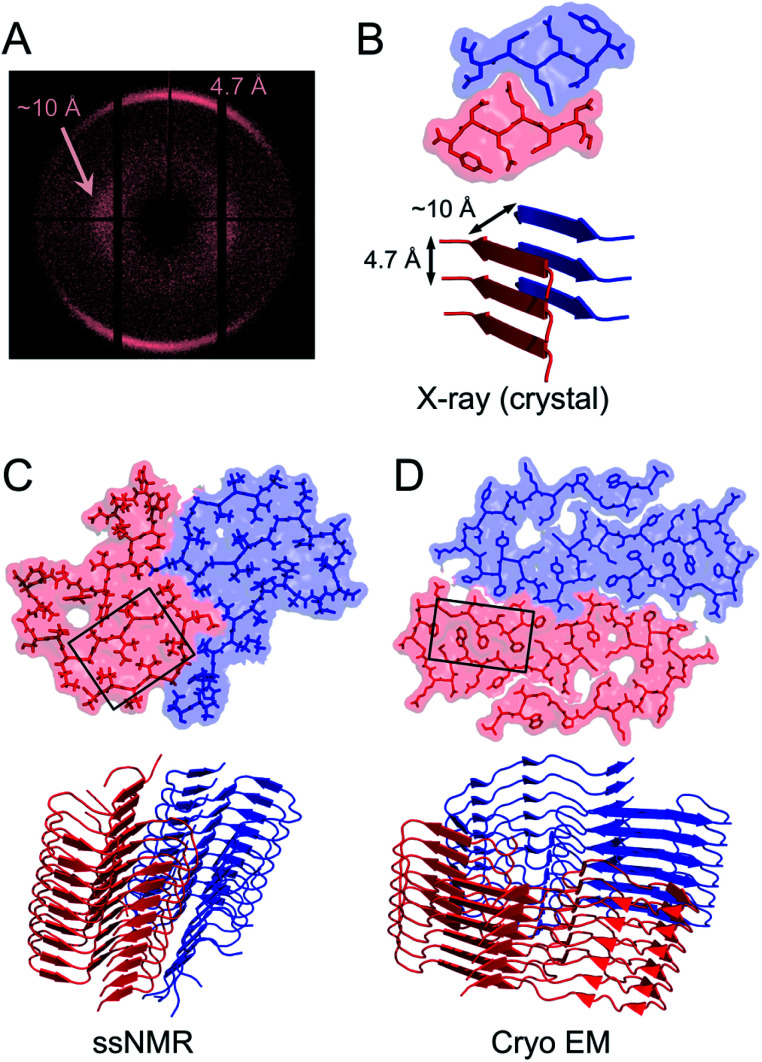
(A) Cross-β wide angle X-ray diffraction pattern. The distances corresponding to the meridional and equatorial reflections are illustrated in (B). (B–D) Comparison of amyloid structures determined by X-ray crystallography ((B) heptapeptide from the yeast prion protein Sup35, PDB ID 1YJP), solid-state NMR ((C) 42 residue Aβ peptide, PDB ID 5KK3) and cryo EM ((D) full-length human serum amyloid A1-protein, PDB ID 6MST). Panel (B) illustrates a “steric zipper” structure while regions with “hetero zippers” are indicated by black boxes in (C) and (D).

X-ray scattering experiments was the first method used to characterize fibrous proteins structures, see for instance the work by Astbury^42,43^ and Pauling.^44,45^ The cross-β structure, that is the structural definition of amyloid-like PNFs, was established in 1968.^46–48^ In this characteristic scattering pattern of axially aligned fibrils meridional reflection at *ca.* 4.7–4.8 Å represents the strand separation within a β-sheet while the sheet packing gives equatorial reflections between 8 and 10 Å (Fig. 3A). This reveals the fundamental architecture of the PNFs: peptide chains that are aligned perpendicular to the fibril axis and assembled into long β-sheets that may pack against each other (Fig. 3).

X-ray crystallography is indeed the most frequently employed method to determine protein structures, but in general it is not applicable to PNFs since they do not form single crystals. However, many short peptide fragments have been found to form microcrystals with packing similar to amyloid fibrils. This has been exploited to determine crystallographic X-ray structures and provide insight about the core structures of amyloid fibrils (Fig. 3). Pioneering work from the Eisenberg lab established the steric zipper model in which β-sheet packing is stabilized with tight interdigitation of the sidechains in a dry interface.^39,49^ More recently an alternative packing mode was discovered for fibrils assembled from low complexity domains. This type of structure that involves close-backbone–backbone contacts between the peptide segments was termed “LARKS” (low-complexity, amyloid-like, reversible, kinked, segments) and has less favourable binding energy than steric zippers.^50^ The steric zipper models suggested a high degree of structural symmetry and self-complementarity of the segments, giving the impression that the amyloid core may consist of “simple” β-sheets with disordered regions surrounding it.

As X-ray crystallography is only suitable for amyloid-like microcrystals, much of the progress in the structural biology of amyloid comes from solid state NMR spectroscopy. The structural models of Aβ1–40 fibrils^51^ and fibrils consisting of the TTR105–115 fragment^52^ based on solid-state NMR data signify the breakthrough of this method. The NMR data for TTR105–115 was later combined with X-ray diffraction and electron microscopy to provide a hierarchical model for a peptide amyloid fibril.^53^ This work shows that even short peptide may form supramolecular structures of non-trivial complexity and illustrate the power in combining different experimental methods. The NMR structures of polypeptides longer than 10 amino acid residues, *e.g.* ref. 54–58 revealed that longer polypeptides assemble into fibrils in a different way than short peptides, with parallel β-sheets as the dominant secondary structure. They form several β-strands with loops or turns between them resulting in “hetero zippers” (Fig. 3).^25^ Instead of providing the cross-β spine, the symmetric intermolecular contact become important for assembly of protofilaments into the mature fibrils. Hence, structures of amyloids from longer sequences suggest that the polypeptide undergo “2D folding”, *i.e.* a simplified version of the 3D folding process for native proteins, where polypeptide chains adopt (one of) the energetically most favourable arrangement(s) on a 2D grid, (sometimes tilted). However, the dominating energetic contribution from the β-sheet intermolecular interactions makes it possible to find different protomer conformations with similar energies, hence molecular polymorphism appears (*vide infra*). Different conformations may also possess different ability to create strong inter-filament contacts, which could also affect the structure of the amyloid.

Structural studies using NMR requires samples with isotopic labelling (^13^C and/or ^15^N). The proteins have to be produced recombinantly using isotopically enriched media or in the case of short-to-medium sized peptides (*e.g.* amyloid β), synthetic peptides with specific isotopic labeling can be used. Hence, both approaches prohibit the use of samples from natural sources. This has been a hurdle for the study of medical amyloid but is of course a problem of the same magnitude for material applications using proteins from less pure resources. Tycko and co-workers demonstrated that it is possible to template and amplify PNFs with specific morphologies by adding synthetic amyloid β peptides to amyloid extracts from Alzheimer patients.^59^ Similar approaches may also be useful for structure determination of PNFs for material applications.^60^

During the last few years, the field has been completely dominated by cryo EM for determination of amyloid structures.^35,61^ The main advantage of this method is that no special requirements, such as crystallinity or isotopic labeling, is needed. The amount of sample needed is also very small and it can handle polymorphic samples as the approach relies on single particle analysis. This has produced an impressive number of high resolution structures (see *e.g.* the list in ref. 35) that open new perspectives on the amyloid structure, not the least from *in*/*ex vivo* patient samples. More intricate 2D folding patterns have also been revealed involving the whole polypeptide chains for larger proteins showing that large parts of a protein can form the fibrillar core, although most fibrils still only have a smaller part of the chain in the core. The variety of structures that are now available has revealed rich variations in stabilizing forces in the amyloid core, including packing of both hydrophobic and polar surfaces, specific hydrogen bonds or salt bridges and co-factors.^32^ This supports that there is a driving force for the protein chain to find an energy minimizing 2D conformation in each fibril layer. Hence, the properties of PNF materials will, to some extent, rely on the amino acid sequence of the protein raw material, which opens for selection/design for specific properties.

### 3.2 Polymorphism of PNFs

The traditional view of protein structure states that each protein has evolved to have a specific amino acid sequence that will be associated with a unique folded structure. The uniqueness of the folded structure comes from the fact that it represents a state with significantly lower energy than any other state. From a molecular perspective, this is achieved by optimal packing of the side chains (primarily the hydrophobic groups) to give a favourable enthalpic term that dominates over the conformational entropy of the disordered polypeptide chain. The change in free energy is roughly proportional to the reduction of the accessible surface area (ΔASA) of the folded structure compared to the unfolded state.^62,63^‡‡ΔASA (non-polar) was initially proposed to be related to the hydrophobic driving force for protein folding.^62^ There is, however, a strong correlation between ΔASA (non-polar) and ΔASA (total).^64^ We here make use of the change in accessible surface area to qualitatively illustrate the principles of polymorphism.

**Fig. 4:**
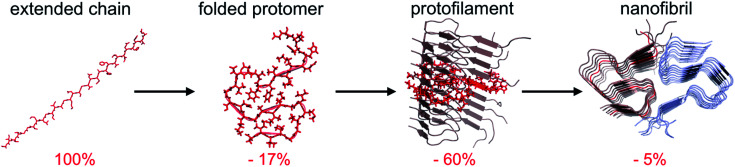
Change in accessible surface area (ASA) as a result of folding of a fully extended chain into a protomer with the conformation adopted in the fibril, insertion of the folded protomer into a protofilament, and association with a second protofilament to form a nanofibril. Average values from 10 different amyloid structures involving 2 or 3 protofilaments.

It has recently become accepted that the native, folded structure is not the global energy minimum. Instead, the amyloid structure (and ultimately amyloid crystals) is the most stable structure a polypeptide chain can adopt.^31^ This is maybe not so surprising considering the fact that the reduction in accessible surface area of a protomer that is incorporated in an amyloid fibrils is typically 80–85% compared to the extended chain (Fig. 4).§§Typical ΔASA values for globular proteins are 60–70%.^64^ However, that should not be directly compared to the number for PNFs since only the segments that form ordered structures are included in the analysis presented in Fig. 4. The major contribution to the favourable energy (reduction of the accessible surface area with 60% compared to an extended chain) comes from packing and hydrogen bonding to the neighbouring protomers in the protofilaments. This process is to a large extent driven by main chain interactions but also packing of side chains. The second largest contribution (*ca.* 15–20%) comes from the “2D folding” of the polypeptide chain when adopting the specific conformation of the amyloid template. On top of this, additional binding energy (only *ca.* 5%) can be gained by twinning two or more protofilaments together. A comparison of ΔASA for these three structural levels provides some clues about the level of variation of the polymorphism at different structural levels. The packing of folded protomers into the protofilament cross-β structure is what provides the major part of the stabilizing energy. Hence, that is the basic feature that is common to all PNFs. The 2D folding only contributes 1/4 to 1/3 of the stabilizing energy, and there will thus be more room for variation in the specific packing (molecular polymorphism). However, the folded 2D-structure will still be of importance, as for many such structures it may not be favourable to pack into protofilaments, and hence many potential 2D-structures will not be tolerated. Finally, the packing of protofilaments into the nanofibril contributes only a small part of the stabilization energy, hence there will be a large variation of the packing polymorphism resulting in an almost continuous distribution of different structures as outlined below.

### 3.3 Nanoscale morphologies

**Fig. 5:**
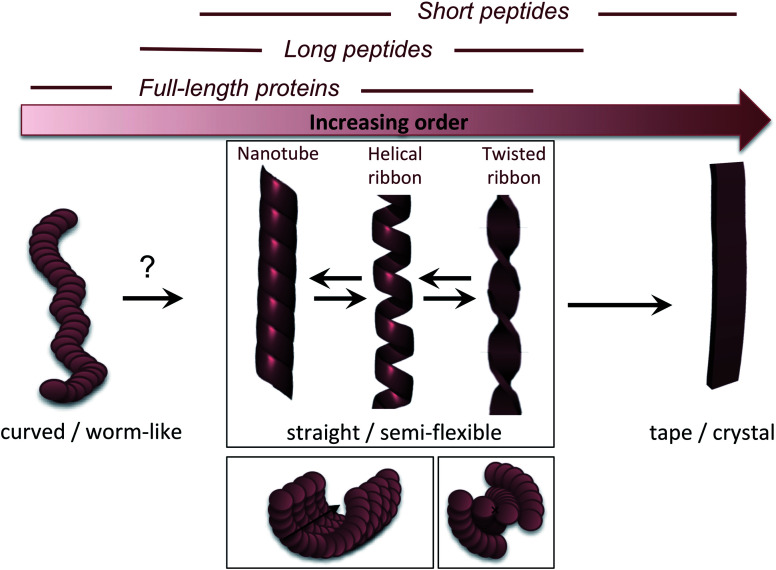
Nanoscale morphologies arranged from low to high order. The typical building blocks (in terms of chain length) that are associated with these morphologies are indicated.

The molecular/conformational polymorphism has been discussed in Section 3.1. The morphology variations at the nanoscale include both the number of filaments and their relative orientation. This, in turn, leads to a rich variation in mesoscopic structures with varying degree of order (Fig. 5). High resolution AFM has, together with EM, provided the basis for understanding the relationship between these structures (see. *e.g.* the review by Adamcik and Mezzenga^31^). Transitions between these types of structures can occur without fibril decomposition. The species traditionally referred to as amyloid-like fibrils are long, straight or semi-flexible nanofibrils (Fig. 5). They are composed of a number of protofilaments that are typically packed laterally in a ribbon like manner.^65^ The ribbons can then adopt superstructures that can be classified into twisted- or helical ribbons.^31,66^ With increasing number of filaments, the helical ribbon structure can close itself and transform into a nanotube.^66^ The twisted ribbons display a periodicity (pitch) that correlates with the number of filaments in the structure, with a longer periodicity for broad ribbons with many filaments.^65^ There is a clear connection between the energy required to bend a fibril and the number of protofilaments making up the fibril, with a larger energy required to deform a fibril made up of many protofilaments, hence there is a connection between the molecular structure and to the mechanical properties of any macroscale material. In the extreme, where the ribbons no longer display any twist, they may turn into microcrystals^31^ (as mentioned above). Amyloid crystals are proposed to be the most thermodynamically stable morphology but as they require ordered packing in 3 dimensions, this remains speculative for longer polypeptide chains; the longest peptide chain for which this type of packing has been demonstrated is a 26 amino acid residues long peptide.^67^ In general, short peptides often display a richer variation in morphology^66–69^ including ribbon structures with different number of filaments as well as more ordered morphologies such as tapes and crystals.^31^ It is also noticeable that short peptide PNFs often involve packing of several molecules in all spatial directions and thereby intrinsically has a more crystal-like architecture.^53,67^

**Fig. 6:**
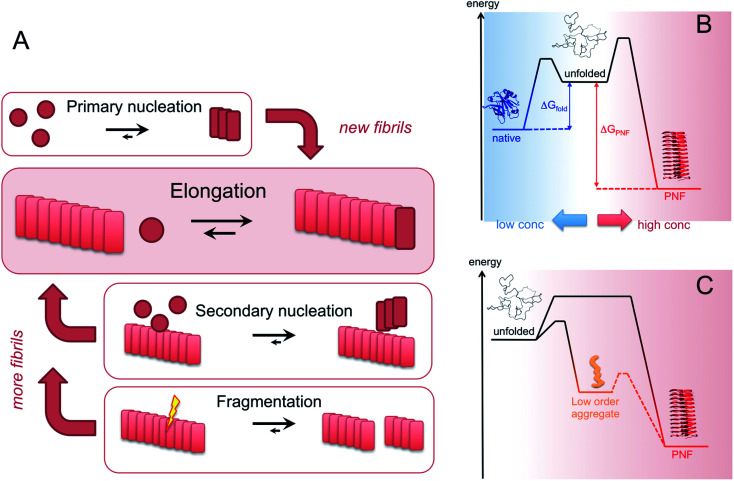
(A) Microscopic processes that leads to the assembly of PNFs. (B) Energy diagrams illustrating the stabilities and kinetic barriers of the native state and the PNF state, respectively. In addition to the energy differences, the chemical potential of the soluble monomer also depends on the protein concentration which must be increased above the solubility limit for the phase transition to occur. (C) The supramolecular part of the diagram with an example of metastable, low order aggregates included. The dashed line path may or may not exist, corresponding to on- or off-pathway intermediates, respectively.

Many attempts of producing PNFs from various industrial/agricultural resources have resulted in fibrils with a curved, worm-like appearance (Fig. 5).^70–73^ Beside their more evident curvature, these fibrils are typically also thinner and they have different nanomechanical properties than the “straight” type of fibrils with significantly lower values of the persistence length and Young's modulus.^74,75^ They also behave differently in the manufacturing of macroscopic, hierarchical materials, illustrating nicely that the nanoscale structure can dictate macroscale properties.^75^ The structural basis of the worm-like fibril morphology is, however, not fully elucidated yet. In some cases (*e.g.* BSA^76^) they may be protofilaments that have not yet assembled into multifilament structures. In other cases they are clearly distinct types of aggregates (*e.g.* β_2_-microglobulin^77^). Their resemblance with what is often referred to as protofibrils of disease-related proteins, *e.g.* amyloid-β,^78^ raises the questions whether they are really amyloid-fibrils or some kind of pre-fibrillar oligomers. For *e.g.* lysozyme or the mouse prion protein, there is a clear connection to smaller oligomers.^79–81^ However, some of these fibrils display typical cross-β patterns in fibre diffraction experiments,^72,82,83^ confirming an amyloid-like architecture. It also seems that worm-like fibrils (at least in some systems) may originate from non-nucleated assembly and thereby represent a kinetic end-product in competition with the thermodynamically more stable fibrils (see Section 4 and Fig. 6C).^77^ Interestingly, a recent paper showed that worm-like fibrils from lysozyme formed at pH 7 dissociated upon heating and reformed again while lowering the temperature, clearly demonstrating a lower thermostability compared to long straight fibrils formed under similar conditions.^83^

The fact that worm-like fibrils often appear for more complex and longer proteins indicate that they may be less ordered at the molecular level. Indeed, for β_2_-microglobulin, worm-like fibrils have been shown to have a smaller amyloid core and larger dynamic regions.^84–86^ Lendel *et al.* noted that curved β-lactoglobulin fibrils formed after protein hydrolysis had a slightly different peptide composition than straight PNFs,^71^ and proposed that the hydrolysis process that precedes the assembly of fibrils (described in more detail in Section 4) may also define the morphology of the formed PNFs. This assumption is strengthen by the fact that synthetic peptides corresponding to the core regions of soy bean PNFs formed long straight fibrils, while the full-length proteins mainly formed curved.^73^ Moreover, the strong connection between the reaction conditions (pH, ionic strength, organic solvent, *etc.*) and the morphology suggest that the intermolecular interactions are important and that worm-like structures may have a lower density of backbone hydrogen bonds.^74^

Based on the available data, at least three different structural models could explain the worm-like appearance of PNFs: (1) modular assembly of oligomeric species giving weaker interactions between the oligomers than within them. It is not clear, however, if this model can still be in agreement with the cross-β structure. (2) PNFs with similar core architecture as straight fibrils but smaller part of the sequence involved in the ordered β-sheet core, *i.e.* larger disordered parts outside the core. This model requires that the final structural conversion of the β-sheet core is associated with a significant energetic barrier to explain the metastability of the worm-like fibrils. (3) A completely different architecture of the β-core, which is faster to form but less stable. Such structure is expected to have a lower degree of intermolecular interaction and thereby also a less ordered structure.

The different nanoscale morphologies have distinct nanomechanical properties that have also been shown to propagate to the micro- and macro scale levels. For a detailed description of the mechanical properties theory we suggest reading the work by Usov, Adamcik and Mezzenga.^31,76^ However, it is obvious that learning how to control the fibrillation pathways and steer it to the type of PNFs with the desired properties (functional or mechanical) will be an essential question to solve in order to fully exploit their possibilities in material science.

## 4. Transformation of proteins into PNFs – from which proteins and how?

Amyloid-like nanofibrils can form under a variety of conditions with large variations in the fibrillation kinetics. The list of proteins that have been confirmed to form PNFs is continuously growing. Some recent summaries can be found in ref. 15, 33 and 87. The same protein can also assemble into fibrils with different structures (polymorphism). The nanomechanical properties are related to the morphology of the PNFs,^74,88^ and also determine the functional and mechanical features of macroscopic materials assembled from them.^75^ Hence, there should be endless opportunities to control and fine tune the properties of PNF-based materials.

### 4.1 How are the nanofibrils formed?

Amyloid-like PNFs are formed through a nucleated polymerization process^89,90^ and is, as all assembly processes, favoured by high monomer concentration. The fibrillar aggregates elongate by adding monomer units at the protofilaments ends. The newly added unit is templated to adopt the same structure (folding pattern) as the unit previously incorporated. The whole process can be modelled by a set of microscopic processes with distinct rate constants^89^ as illustrated in (Fig. 6A). Initiation of the process requires a primary nucleation event in which the very first nuclei form. The nucleation can be homogenous (from only monomers in solution) or heterogeneous (catalysed by other components, *e.g.* membranes, phase interfaces). The homogenous nucleation step is a low probability process that passes a free energy barrier, and can be circumvented by added pre-formed amyloid fibrils or fibril fragments (seeding). As soon as the first nuclei are formed, additional growth is thermodynamically downhill, resulting in rapid growth of PNFs (*i.e.* elongation of protofilament). Fibril growth is an autocatalytic process where preexisting fibrils accelerate the conversion of monomer, in a process known as secondary nucleation where the surface of already existing fibrils act as nucleation sites. In addition, fibril breakage can further increase the growth rate, as this creates more ends where elongation can occur. The balance between these processes has been thoroughly investigated for *e.g.* amyloid-β and these studies particularly highlight the secondary nucleation process as critical for the typical sigmoidal growth curve (Fig. 2).^91–93^ On the other hand, functional amyloids, like those in bacterial biofilm, seems to form in the absence of secondary processes and self-amplification, which may be a way for the organisms to keep the process under tight control.^94^ Little is so far known about the assembly mechanisms of artificial, materials-related PNFs and the molecular determinants of the different assembly pathways.

The process by which a protein monomer in solution is incorporated in a PNF can be described as a phase transition.^95^ As for any other phase transition, the chemical potential of the molecules in any of the existing phases will be the same at equilibrium conditions. In a simplified model with only two states (monomer in solution or attached to fibril), the amount of polypeptide molecules in the fibril phase at equilibrium (*i.e.* the total number of molecules minus the amount of molecules in solution) is determined by the stability of the amyloid state (*i.e.* the difference in standard chemical potentials between a molecule incorporated in a fibril and a monomer in solution).^95^ At equilibrium, the more stable the amyloid state is, the lower the concentration of soluble monomers will be. Although this description only apply strictly to the simplified model, the stability of the amyloid state relative other states will determine if PNFs are formed, what peptide concentration that is needed and how much fibrils that can be obtained (provided that the system can reach equilibrium). In reality, kinetic barriers originating from folded native monomers or alternative, less ordered aggregates (*e.g.* worm-like fibrils as described in the previous section) will be important for the possibilities of transforming a protein into amyloid-like PNFs (Fig. 6B and C).

For most proteins the amyloid state is to be considered the thermodynamic ground state and the native, “correctly” folded structure only represents a metastable state.^31,96–98^ However, the native structure folding equilibrium will not have a concentration dependence, as the molecular interactions are intramolecular, and therefore dominate at low protein concentrations. Once formed, the native structure may be stable enough to provide a significant kinetic barrier for transformation into a fibrous state, in addition to the barrier associated with the low probability nucleation event.

As the structural features of the native and PNF states are different, they also display different thermodynamic signatures. Baldwin *et al.* described that, unlike for native protein structures, there is a clear correlation between the chain length and the stability of the amyloid state (measured as Δ*G* of elongation) that is only weakly affected by the amino acid composition.^97^ There is also a maximum stability (minimum Δ*G*) at a polypeptide length of *ca.* 100 residues, suggesting that longer chains may lead to structural frustration and thereby reduce the thermodynamic stability of the PNF state.^97^ Hence, it is likely that really large proteins will not be able to form amyloid without prior processing into smaller segments. It should also be noted that the value of 100 amino acids chain length is obtained from a biased set of known amyloidogenic proteins, meaning that the peptide chain in these proteins is able to fold into a 2D-structure that can efficiently be incorporated into a protofilament. For a non-biased sample of polypeptides with a random amino acid sequence, the propensity to form PNFs is likely higher the shorter the chain (although a longer chain that can be fully incorporated in the fibril structures most likely gives a larger stabilization compared to a shorter chain). This is also evident in the literature, as the assembly of short peptides into fibrils is frequently observed while large proteins require more harsh conditions.

**Fig. 7:**
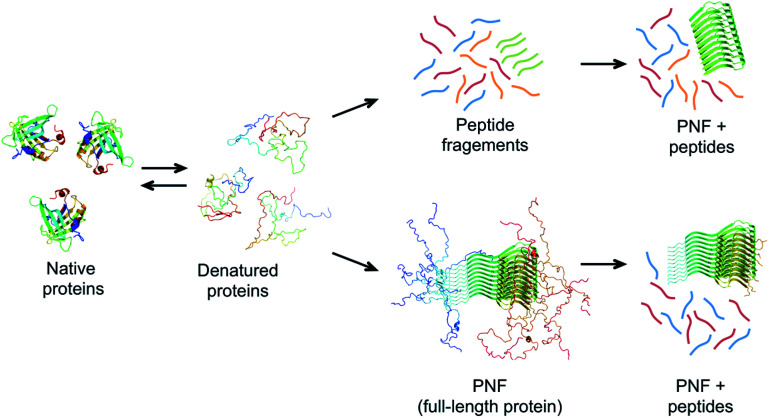
Possible mechanisms of PNF-formation at hydrolysis-promoting conditions. The protein must be (partially) denatured to form fibrils. Chains with high propensity to form amyloid structure may form PNFs from the full-length protein (lower pathway). These fibrils typically do not have the whole chains in the core and disordered parts could be cleaved off after PNF formation. An alternative route involves hydrolysis of the protein chain into peptides before assembly (upper pathway). Some of the peptides may have high propensity to self-assemble into nanofibrils.

Based on these principles, a prerequisite for the assembly to proceed within a reasonable time frame is that the protein can access a fibrillation-competent state. A stably folded protein is well protected against “misfolding” and aggregation. Hence, to initiate PNF formation the protein may need to be subjected to conditions that destabilize the native state, by a change in temperature or chemical environment. Denaturing additives (urea, GuHCl) or organic solvents (*e.g.* alcohols) will alter the energies of all states, typically in favour of more disordered structures. Since the amyloid state has a lower energy to start with (stronger interactions) it can tolerate more denaturants than the native state and will still be energetically favourable at reasonably high denaturant concentrations. Structural destabilization can also be achieved by high temperature and pH changes. Several different proteins have been reported to form nanofibrils when incubated at low pH (below 3) and high temperature (50–90 °C).^99–106^ Besides reducing the stability of folded proteins, the generic fibrillation-promoting nature of these conditions is partly related to the uniform positive charge of the protein molecules at extreme pH values below their isoelectric points.^107^ This causes repulsive forces between the molecules and prohibits unspecific aggregation. Only highly optimized structures, such as the amyloid cross-β, can provide enough binding energy to overcome the charge–charge penalty. The strength of the repulsive forces can also be altered by changing the ionic strength of the solution. Increased salt concentration leads to electrostatic screening that lowers the energy barrier for assembly but also sometimes allow the formation of less ordered fibrils.^107^ The high temperature speeds up the assembly reaction but, more importantly, at low pH it also promotes hydrolysis of the protein into smaller peptides that may assemble in an ordered way more easily than the full-length protein (Fig. 7).^71,108^ The link between hydrolysis and fibril formation has been shown for several proteins.^72,73,103,109–111^ Notably, proteolytic cleavage of proteins is commonly observed for diseases-associated amyloid proteins.^21^

The role of hydrolysis can be rationalized in the light of the length-dependence of the amyloid stability described above. Some amino acid segments have higher propensities to form β-sheets and amyloid than others and the stability of an amyloid from a longer chain will be a trade off between the parts that form ordered β-sheet structure and the parts that remain disordered (Fig. 7). This can be seen in some amyloid structures, where the cross-β core only consists of parts of the full-length protein, for example, in the structure of α-synuclein fibrils,^56^*ca.* 47% of the protein is located in the core β-sheets. Therefore, the fibrils formed from the shorter peptide chains originating from hydrolysis will have a more favourable free energy than the full-length protein. In fact, hydrolysis will completely reorganize the energy diagram in Fig. 6. The conformational entropy of the disordered monomer is reduced (because it is shorter) and the native, globular state may no longer exist. Based on this line of arguments, any post-fibrillation hydrolysis of non-core, unstructured protein segments (“shaving”)^112^ would also increase the stability of the fibrils (Fig. 7). With this in mind, it seems likely that hydrolysis is the main explanation that many large and complex proteins can be convinced to form PNFs at low pH and high temperature. Noticeably, this will also mean that the yield of PNF formation will never be 100% (based on the mass of the starting material) since not all peptides will form stable PNFs.

**Fig. 8:**
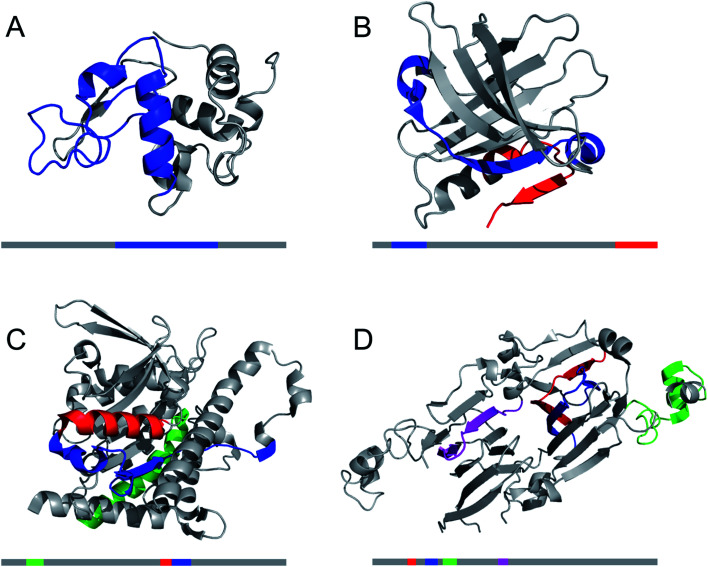
The structural properties of PNF-forming peptides in the native structures of the proteins they originate from. The grey bars below the structures represents the protein sequences with the peptide positions indicated in the same colours. (A) Hen egg lysozyme (residues 53–100,^113^ PDB ID 1JPO). (B) Bovine β-lactoglobulin (residues 12–32 and 138–162,^109^ PDB ID 2Q2M). (C) Patatin from potato (residues 36–60, 224–240 and 247–266,^72^ PDB ID 4PK9). (D) Soybean glycinin and β-conglycinin. The protein subunits have high structural similarity and peptides from different subunits are illustrated in the structure of β-conglycinin subunit β.^73^

A large number of peptides segments originating from amyloid-forming proteins have been investigated with respect to fibril formation. The most remarkable results are probably the trimming of a segment in the amyloid-β peptide (…KLVFF…) down to a diphenylalanine peptide that was found to form nanotubes.^8^ A few studies have investigated what peptide segments that form the amyloid cores when full length proteins are incubated under hydrolysis-promoting conditions. Such studies provide a glimpse of what structural segments most easily nucleate PNFs and could potentially be used to seed reactions. Fig. 8 illustrates some examples of globular proteins that have been investigated in detail, including peptides from egg lysozyme,^113^ milk β-lactoglobulin,^109^ patatin from potato^72^ and soybean proteins.^73^ One interesting detail that can be observed is the two segments in β-lactoglobulin that are in different ends of the sequence but actually constitute the strands next to each other in the native β-sheet structure. This may be important for initializing the fibrillation process. Another fascinating finding is the three different segments from soy proteins that constitute ‘β-archs’ motifs^114^ that have obvious similarities with the molecular structures of protofilaments. However, looking at all the examples, there are no obvious structural preferences of the segments in the native state as α-helices, β-strands and random coil structures are all represented. There is also a variation on the positioning of the segments within the full-length sequences with both middle segments (*e.g.* lysozyme) and termini (β-lactoglobulin) represented. One potential trend is that the segments become shorter in larger proteins, suggesting that hydrolysis becomes more important to initiate the fibrillation process.

### 4.2 Which protein sources can be used for PNF materials?

The amyloid state, previously regarded as a medically peculiar misfolded state is now recognized as a generic structural form of most, if not all, proteins.^115^ Taking a random protein and running the sequence in an amyloid prediction algorithm, through utilization of statistics and/or the physicochemical properties of the amino acid sequence, likely will identify one or a few fibrillation-potent regions. In 2010, Goldschmidt *et al.* predicted that basically every protein (99–100% of the proteins coded by the *E. coli*, *S. cerevisiae* or *H. sapiens* genomes) contains at least one segment with high propensity to form amyloid structures.^116^ This finding predicts that a wide range of proteins should be able to form amyloid, in correspondence with the variety of different proteins which have already been confirmed to form PNFs.

This finding raises the question, why our cells are not full of amyloid fibrils? One explanation is the protective role of the native structure described above. There is also a need to keep the concentrations of protein as low as possible. In addition, there seems to have been an evolutionary driving force to hinder the proteins to “misfold” into amyloid structures (because that would destroy their functions).^117^*In vivo* there is also extensive quality control machinery with chaperones that help the proteins to keep their native functional structures. Functional amyloid structures must be assembled in a controlled way to avoid runaway aggregation.^94^ This is why rare events (such as mutations in the protein sequence) or the reduced efficacy of the quality control mechanism during ageing are necessary to trigger disease-associated amyloid formation in living organisms. However, when exploring the amyloid formation for engineering of materials, we are not limited to physiological conditions. We can use whatever means necessary to open up and destroy the protective native structures and direct the proteins into their more stable fibrillar states.

Outside the medical field, one of the most frequently studied PNF system today is the fibrils formed by the bovine whey protein β-lactoglobulin.^22,118,119^ PNFs can easily be produced in gram- or even kilogram scale at a reasonable material cost from whey protein isolate (WPI). Despite the fact that WPI is a crude mixture of proteins and the second most abundant protein (α-lactalbumin) also have been shown to be able to fibrillate,^120^ the PNFs from WPI are primarily formed by β-lactoglobulin.^71,121^ Among the reasons why β-lactoglobulin is popular is its small size and high solubility (at least 9 wt%).^122^ Solubility is an important aspect since insoluble protein is kinetically trapped in alternative aggregates. The solubilization of protein raw materials in aqueous solvents is indeed a recognized challenge for many of the proteins that could constitute the basis for production of sustainable materials.^123^ Note that this solubility refers to the ability of protein powder to be dissolved in water. This solubility is different from the earlier mentioned solubility describing the equilibrium propensity of a monomer to remain in solution phase relative to be incorporated into the amyloid phase. This latter solubility is expected to be lower than the solubility of the protein powder, since the amyloid is the thermodynamically more stable state. Indeed, for β-lactoglobulin the “critical aggregation concentration” was measured to *ca.* 0.2 wt%.^124^ The work on β-lactoglobulin illustrate many of the general features of PNF formation: (1) promotion of fibrillation by denaturants,^125^ organic solvents such as alcohols^106,126^ or low pH and high temperature;^106,126^ (2) changes in fibrillation kinetics and fibril structure by factors such ionic strength,^127–129^ additives,^125,126,130,131^ heating mode^132^ or mechanical processing;^133^ and (3) the importance of peptide hydrolysis for PNF formation at low pH.^71,108,109,134^

Other animal derived proteins that have been explored for PNF formation are: hen egg lysozyme,^135,136^ egg ovalbumin,^137^ bovine serum albumin,^138^ milk casein,^139^ bovine insulin,^30^ fish eye crystalline^140^ and bovine hemoglobin.^141^ Although the preferred protein source depends on factors such as availability, processing equipment and the intended application, plant proteins are often more attractive than animal proteins from both an ethical point of view and a sustainability perspective. However, compared to the extensive research on disease-related amyloid and animal-derived model systems, such as β-lactoglobulin or lysozyme, the knowledge about the fibrillation mechanisms of plant proteins is limited. Nevertheless, plant proteomes are predicted to contain similar levels of amyloidogenic protein sequences as *e.g.* human- or *E. coli*-proteins.^142^ Fibrillation *in vitro* has so far been reported for several plant proteins, mainly from legumes but also *e.g.* rapeseed, oat, and potato proteins.^70,72,99,102,103,105,111,143–149^ However, most of the studies so far do not provide detailed mechanistic understanding. Interestingly, a recent study provided the first example of a functional amyloid in plants, as it showed that seed storage proteins in peas may adopt a functional amyloid stage to facilitate storage and potentially also as a microbial defense.^26^ Hence, there may be an inherent amyloid propensity of plant seed storage proteins which could be very useful for technological applications as they are the most abundant proteins in plants. As more groups are now starting to work with these systems it is expected that this knowledge will increase over the next couple of years and hopefully reveal common trends that can help the selection of protein raw material for specific applications.

The increasing list of proteins that can form PNFs opens the possibility to utilize any protein raw material for the development of new materials. Finding the appropriate condition for converting the protein into PNFs (with energy efficiency still in mind) can, however, be challenging. As described above, the protein powder should be solubilized and the native structure must be destabilized. The realization of the role of hydrolysis may facilitate this process, as low pH conditions are often found to be good for PNF formation. With increasing knowledge about what type of peptides that forms the most stable fibrils more sophisticated methods could be employed, such as selected enzymes to produce high concentrations of specific fragments. This could both improve the rate and the yield of the process as well as allowing for controlling the morphology of the PNFs.^150–152^

## 5. Functionalization of PNFs

If materials are classified according to manufacturing complexity (*i.e.* their ease of production) and cost of production it can be stated that the value of high complexity materials resides in the information content required to produce it, whereas the value for low complexity materials primarily resides in the material itself. In other words, if proteins can be organized into particular structures enabling valuable functions, the cost of the end-product is no longer simply related to the cost of the starting materials.^19^ In addition to the possibility of acquiring novel properties for the protein itself through processing it into new structures (such as PNFs), the wide range of functional groups and structural elements displayed by proteins can provide favourable interactions with various reagents and thereby possibilities to functionalize the protein. A spectacular example from nature is the incorporation of chromophores to the photosynthetic proteins, that enable harvesting of light. Examples involving PNFs are vehicles for drug delivery^153^ or light emitting molecules.^154^ Early studies on PNF functionalization also investigated the amyloid structure as a template for formation of inorganic nanowires, as demonstrated by Scheibel and co-workers and others.^8,155,156^ Another fascinating example of the functionality inherent in PNFs themselves are their efficiency as scavengers for metal salts and organic pollutants, allowing purification of contaminated water.^157–159^

While the specific details will depend on the particular system investigated, we will aim to provide some general guidelines in this section both with regards to functionalization methodology and modes of binding of functionalization agents to PNFs. The possibility to detect amyloid by staining tissue with dyes is one of the most recognized features of the amyloid structure and still widely used in pathology. Many studies have addressed the modes of binding to amyloid by dyes such as Congo red or thioflavin T, or other more recently developed optical probes.^160,161^ However, in a similar manner to the situation regarding amyloid structure, most studies focus on fibrils related to diseases. From a materials science perspective, amyloid staining procedures can be viewed as a functionalization process, where the functionality of the amyloid structure is enhanced by complexation with dyes modifying the optical properties of the material. In the discussion below we will focus on functionalization of amyloid with various conjugated molecules, such as dyes and luminescent molecules, but it should be noted that many other types of functionalization agents can be used, for example drug molecules or nutrients.

### 5.1 General properties of PNFs related to functionalization

**Fig. 9:**
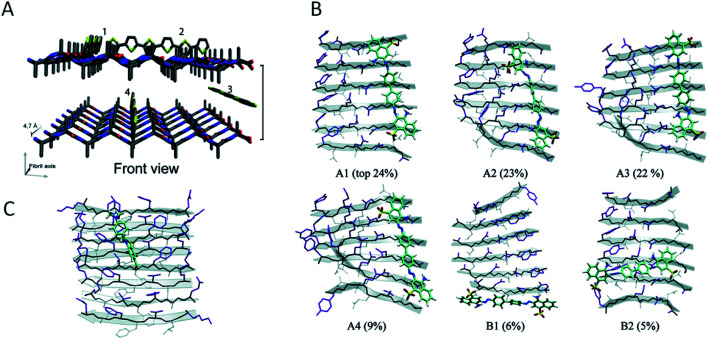
Modes of binding of dyes to PNFs. (A) A generalized fibril fragment with possible generalized types of binding modes for dyes. Adapted from ref. 196 with permission from The Royal Society of Chemistry. (B) Binding modes, according to MD simulations, of Congo red to a GNNQQNY fibril fragment. Different binding modes and their relative populations in % are indicated (adapted from ref. 164. Copyright (2007) American Chemical Society). (C) The dominating binding mode, according to MD simulations, of thioflavin T to a KLVFFAE fibril fragment (adapted from ref. 164. Copyright (2007) American Chemical Society).

Investigations of the interactions between amyloid-specific dyes and amyloid-like nanofibrils have provided valuable information also from the perspective of PNF-functionalization. The main concepts required for a discussion of this topic are illustrated in Fig. 9. The extended β-sheets of the amyloid structure have hydrophobic channels where dyes may bind. The PNFs will also display a highly repetitive pattern of charged groups and groups available for hydrogen bonding that will be important for defining the binding modes of specific molecules. The presence of hydrophobic channels can be viewed as a generic property of the amyloid structure, whereas possibilities for electrostatic interactions or hydrogen bonding will depend on the specific nature of the protein. The presence of extended channels with hydrophobic character means that linear rigid organic molecules bind strongly to amyloid. In dyes for amyloid staining the backbone is typically made up of conjugated π-electron systems, rich in aromatic rings that gives the dyes a structure matching with the structure of the hydrophobic channels in the extended β-sheet. Fig. 9B shows different binding modes, according to molecular dynamics (MD) simulations of Congo red interacting with a peptide fibril fragment. The percentages refer to the population of the different binding modes. It can be concluded that the most common orientation of the Congo red molecule is with its long axis parallel to the long fibril axis, but with a small population of molecules bound perpendicularly to the long fibril axis. Fig. 9C illustrates the dominating binding mode (according to MD simulations) of thioflavin T to another peptide fibril fragment. Even though a different peptide segment and a different molecule is employed, the binding mode is rather similar to that of Congo red, illustrating that as a general rule linear organic dyes prefer to bind with their long axis parallel to the long fibril axis.

Moreover, as described in Section 3 the conversion of a protein into fibrils may lead to polymorphism, where each of the different structures may show different physical properties including different affinity or binding sites for dyes. The widespread occurrence of polymorphism in PNFs is both a challenge and an opportunity: selective preparation of particular morphologies provides an opportunity for fine tuning PNF properties; on the other hand, polymorphism may lead to statistical distributions of structures all having slightly different properties. Another problem related to polymorphism is that PNF formation may be sensitive to small variations in preparation conditions, that may lead to variability between different batches of materials. At present, new amyloid staining dyes are being developed that show different optical signatures upon binding to structurally different amyloid motifs.^162,163^ From a functionalization perspective this may point to opportunities where a PNF structure could be designed to induce desired photophysical properties of *e.g.* chromophores.

In addition to hydrophobic grooves, PNFs will contain a plethora of functional groups (*e.g.* R–SH; R–OH, R–COOH, R–NH_2_) that could be used as handles for functionalization. The exact composition will depend on the particular protein and, in the case of hydrolysis, on the particular sequence of amino acids incorporated into the PNF structure. The specific positioning and accessibility of suitable amino acid residues will also be important, as the functional groups must be reactive enough and be located at accessible positions. In the present text we focus on functionalization by organic molecular materials, and refer the reader to a recent review for a more comprehensive discussion of inorganic systems.^165^

### 5.2 Approaches for functionalization by organic molecules in solution

**Fig. 10:**
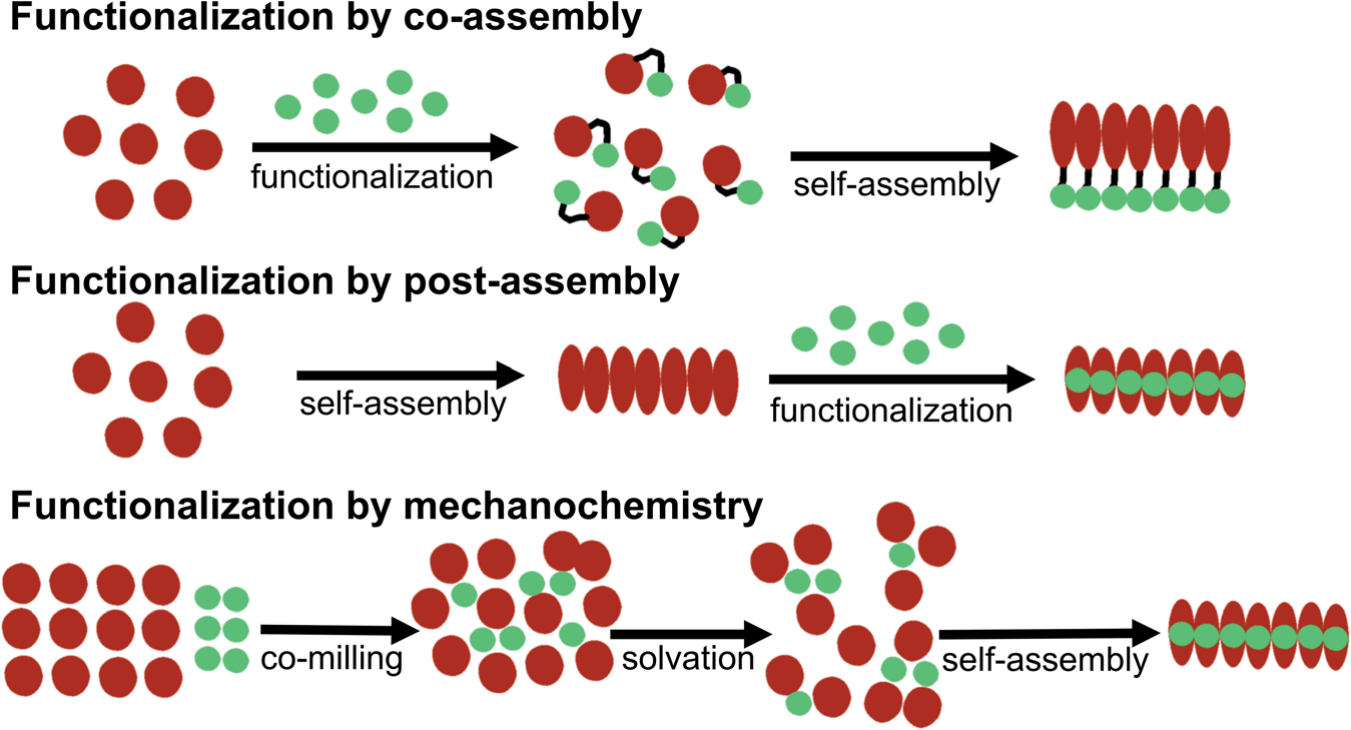
Schematic overview of different methodologies for functionalization of PNFs.

Some basic limitations on reagents for traditional functionalization processes of proteins are related to the common use of water based solvents. The functionalization agent has to be soluble in the same solvent system as the PNFs. As described by *e.g.* Woolfson and Mahmoud^166^ functionalization (they used the term decoration) of self-assembled fibrous biomaterials can be divided into two families of methodologies: (1) co-assembly of covalently linked structural and functional moieties; and (2) post-assembly functionalization (Fig. 10). In addition, hybrid methodologies can be considered, where protein molecules and functional molecules are mixed prior to assembly, the two components can then can undergo co-assembly. Below we divide the discussion of different solution based methodologies into the two families described by Woolfson and Mahmoud, and introduce a third category where the mixing process is performed by milling in the solid state (mechanochemistry).

#### 5.2.1 Co-assembly approaches

This approach relies on molecules where the self-assembling component and the functional moiety are connected by covalent bond conjugation. Such monomers can be prepared by synthetic biology as fusion proteins or by organic chemistry approaches. The functionalization step is accordingly performed before self-assembly, meaning that the presence of the functional moiety may perturb the self-assembly process. The attachment of the functional moiety to the self-assembling moiety can be performed by various conjugation techniques;^167^ however, in the case of proteins there will be limits regarding reaction conditions and solvents that can be tolerated by the relatively sensitive protein molecule. However, if suitable coupling partners are available this is an excellent method for functionalization as the density of functional units can be tuned by combining the same fibrillating protein with and without the functional unit attached.^168^ It should also be noted that often extensive synthesis (chemical or biotechnological) has to be performed before the key step of conjugating the functional moiety to the protein can be carried out, meaning that this methodology can be challenging to carry out on large scale. Coupling partners may be commercially available, but at a prohibitive price that rules out applications in large scale. Often instalment of a spacer will be required in order to allow coupling and this will typically require several additional steps of organic synthesis, before the functional moiety possesses a suitable reactive site enabling conjugation to the protein. Covalent conjugation moreover requires the presence of accessible reactive groups on the self-assembling unit (the protein) by which the functional moiety can be attached. There are several examples of this methodology using short synthetic peptide that after conjugation are assembled into fibrils.^169–172^ These methods should in principle be extendable to proteins capable of forming PNFs although a synthetic biology strategy may then be more attractive than organic chemistry modifications.^173–178^ Another complication is if the PNFs formation rely on hydrolysis of the protein prior to PNF formation. However, with proper knowledge and control of the process, a hydrolysis-related mechanism would in principle allow for combining large-scale protein resources with functionalized peptides (corresponding to the fibrillating regions in the proteins) to form hydrid PNFs.

#### 5.2.2 Assembly of the protein into PNFs in the presence of the functionalization agent

In the hybrid approach mentioned above, the protein to be fibrillated is dissolved together with the functionalization agent. The protein is then exposed to conditions promoting fibrillation, and if the presence of the functionalization agent does not perturb the fibrillation process PNFs will form. An example of this type of process is the assembly of protein to PNF in the presence of a conjugated oligothiophene.^179^ Possible complications can thus arise if the functionalization agent is able to inhibit formation of PNFs. Such a molecule may then be of interest as an inhibitor of amyloid formation.^180^ The presence of the functional molecule could also affect the assembly mechanism resulting in different structural properties of the PNFs compared to fibrils formed in the absence of that molecule. Hence, this could be a way to control both the structural and functional properties of the PNFs.

#### 5.2.3 Post-assembly approaches

The third option is to attach functional groups, by covalent chemistry, onto the preformed fibril. This approach has for example been employed to enable imaging of fibrils growth^181^ and so-called click chemistry has been employed to attach a wide variety of functionalities to amyloid fibrils containing alkyne functional groups.^182^ In a different approach, biotinylation of preformed fibrils allowed functionalization by fusion proteins (*e.g.* enzymes) carrying a streptavidin unit.^183^

In addition, supramolecular (non-covalent) approaches, analogous to the methodology for staining of amyloids with dyes, can be employed. This approach requires that the PNFs can be mixed with the functionalization agent in a common solvent, and that favourable interactions between fibril and the functionalization agent will lead to binding of the functionalization moiety and the self-assembled structure.^184–187^ Since the solvent is often water, these interactions primarily rely on hydrophobic contacts, which is highly suitable for substances that can introduce new photophysical properties or electrical conductivity in the PNF material. To adjust the process to a PNF-friendly solvent, typically either the molecule will contain charged groups rendering it soluble in water, or co-solvents will be employed that are miscible with water. The molecule can then be dissolved in the co-solvent, and this solution be added to the PNF dispersion. Post-assembly functionalization is a common method for functionalization of PNFs. A variation on this theme is polymerizations employing the fibril as a template. This approach has also been employed to form hybrids between PNFs and conjugated polymers, by polymerization on the fibril.^188,189^ The monomer is mixed with PNFs and the mixture is then exposed to conditions promoting polymerization. Unsubstituted conjugated polymers have a low solubility even in organic solvents so the formed polymer will adhere to the PNF template.

### 5.3 Mechanochemical methodology

A large portion of available organic molecules is hydrophobic having a low solubility in water. This creates a practical mixing problem making straightforward functionalization in the liquid phase challenging. However, in the absence of solvent a hydrophobic and hydrophilic material can be mixed simply by co-milling. The act of milling components in order to mix them has no doubt been used by humans since prehistoric times, for example as a way of preparing some of the paints used in pre-historic cave paintings. Reducing the need for solvent is also in line with a more sustainable process. During milling, mechanical forces lead to structural transformations in the materials allowing them to mix. The use of mechanical force to enable chemical transformations is known as mechanochemistry. Nowadays, mechanochemistry is an interdisciplinary subject which involves mineralogy, inorganic and organic synthesis, tribology, polymer science, and biochemistry.^190–192^ Much of recent research focuses on covalent bond formation and formation of co-crystals, due to the possibility of avoiding the use of organic solvents. The use of grinding of a hydrophobic dye material with biopolymers in order to prepare aqueous dye dispersions is well known. However, grinding of insoluble dyes with a biopolymer in combination with solution phase self-assembly is much less explored. In addition to grinding by hand with a mortar and pestle, the process can easily be automatized and scaled up with a variety of grinding/milling equipment is available.^193,194^ In this manner the milling conditions can be systematically modified. In addition to shaker mills, planetary ball mills can be employed that enable scale up and is thus an excellent choice for large-scale chemical processing.

**Fig. 11:**
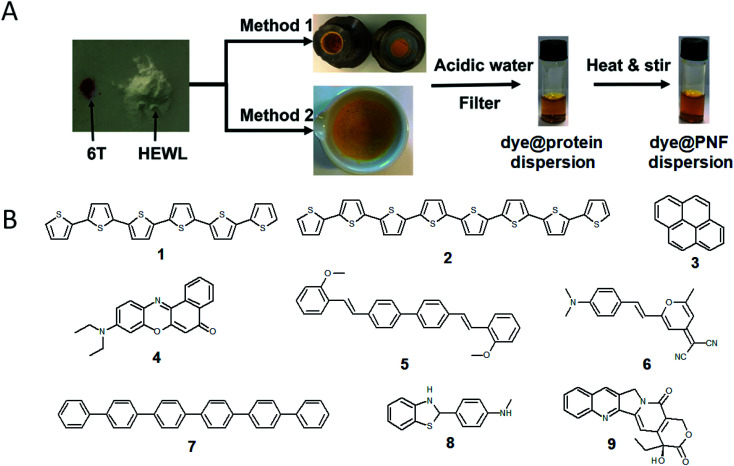
(A) Schematic illustration of mechanochemical methodology to form functionalized PNFs. Method 1 shows an example employing milling cups for use in an automated shaker-mill. Method 2 shows and example where milling was performed by mortar and pestle adapted from ref. 195 with permission from Springer Nature; (B) chemical structures of dyes employed for preparing functionalized PNFs.

Solin *et al.* have developed a methodology for preparation of functionalized protein fibril structures by mechanochemistry, illustrated schematically in Fig. 11. Here the fact that proteins and hydrophobic organic molecules have orthogonal solubility is utilized constructively. As the mixing of the protein and the hydrophobic material is done by co-grinding in the solid state, the hydrophobic material is spread out in the protein matrix, resulting in a hybrid material. Upon addition of water, this hybrid material dissolves, and the hydrophobic effect leads to the burial of aggregates of the hydrophobic materials within micelle-like structures formed by the protein. When heated in aqueous acid the protein still unfolds, and rearranges into structures capable of self-assembly into protein fibrils. As the hydrophobic material is insoluble in water, it will stay associated with the protein during the self-assembly process, thereby ending up in the final fibril structure. A variety of hydrophobic molecules can be employed as exemplified in Fig. 11B where chemical structures are shown for selected molecules/dyes 1–9. These types of molecules are convenient to employ as they have characteristic absorption and emission spectra that facilitates characterization of the materials; however, the methodology also works for other types of hydrophobic molecules/materials. Compounds 1–3 and 7 are examples of extremely hydrophobic aromatic compounds, and compounds 4–6 are examples of laser dyes with low solubility in water. Compound 8 is a hydrophobic ThT analogue and compound 9 is the anti-cancer drug camptothecin. One concern when employing the above methodology is that the hydrophobic molecule might be incorporated into protein fibrils as unordered aggregates. This was investigated by Bäcklund and co-workers for PNF formation of insulin milled with α-sexithiophene (6T).^196^ The emission of 6T is very sensitive to the aggregation state of the molecule, and when a solution of insulin ground with 6T is exposed to fibrillation conditions, dramatic changes in the emission spectra of 6T can be observed as the reaction progresses. AFM images taken at different reaction times demonstrate that the spectral changes are associated with the formation of amyloid fibrils. The results indicate that the state of aggregation of the oligothiophene changes upon formation of amyloid materials. The oligothiophene is initially present as small aggregates surrounded by protein. However, the formation of protein fibrils results in the formation of extended β-sheets with hydrophobic channels into which 6T molecules can disperse. This leads to an overall preferred orientation of 6T along the fiber-axis, as verified by flow linear dichroism (LD) measurements.^196^ These results indicate that even though prepared by a mechanochemical route, small flat hydrophobic molecules will bind to amyloid fibrils in a similar fashion to thioflavin T, Congo red, and water soluble thiophene derivatives. A wide range of material combinations can be prepared utilizing the mechanochemical approach to PNF functionalization. PNFs have been functionalized with phosphorescent metal complexes,^197^ as well as a laser dyes such as Nile red and DCM.^195,198,199^ It should be noted that the presence of the hydrophobic material may dramatically perturb the self-assembly process, resulting in novel structures. However, by employing means such as agitation or stirring the reaction can be directed towards fibril formation.

### 5.4 Multiple functionalizations

**Fig. 12:**
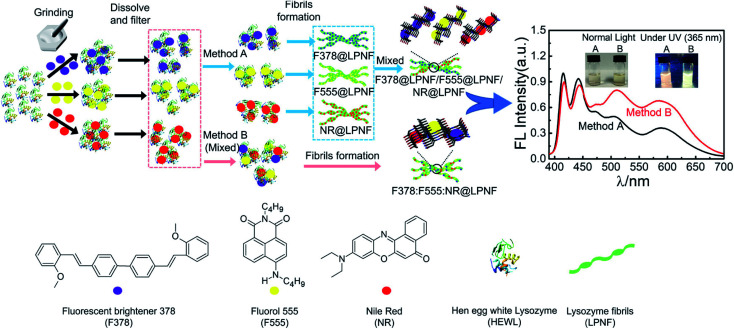
Mechanochemical methodology for preparation of triple-functionalized PNFs giving materials that can convert UV-light to white light through a FRET-cascade. The degree of FRET can be modified depending on if the three types of dyes@protein dispersions are mixed (method B) or not (method A) prior to PNF formation. Reproduced from ref. 203. Copyright (2021) American Chemical Society.

An interesting possibility is to employ the PNF structure as a template where different functionalization agents can be co-assembled. Gorbenko *et al.* investigated FRET in PNFs functionalized by water-soluble donor and acceptor pairs.^200–202^ Yuan and Solin employed the mechanochemical methodology to prepare triply functionalized PNFs, where the three dyes functioned as a Förster Resonance Energy Transfer (FRET) system that could convert UV-light to white light (Fig. 12).^203^ By modifications of the conditions for PNF formation it was possible to influence the extent of FRET. Mixing three types of PNFs, each functionalized with one type of dye, gave a different result than preparing fibrils from a protein–dye mixture containing all three dyes, as illustrated in Fig. 12. It was found that for identical dye ratios, materials prepared by the second method (B in Fig. 12) showed more efficient FRET compared to PNFs prepared by method A. It was moreover possible to influence the extent of FRET, and thus tune the colour of the emitted light, by varying the relative ratio of the different dyes. The FRET system could also be employed as coatings for commercial UV-LEDs.

## 6. Assembly of PNFs into macroscopic materials

**Fig. 13:**
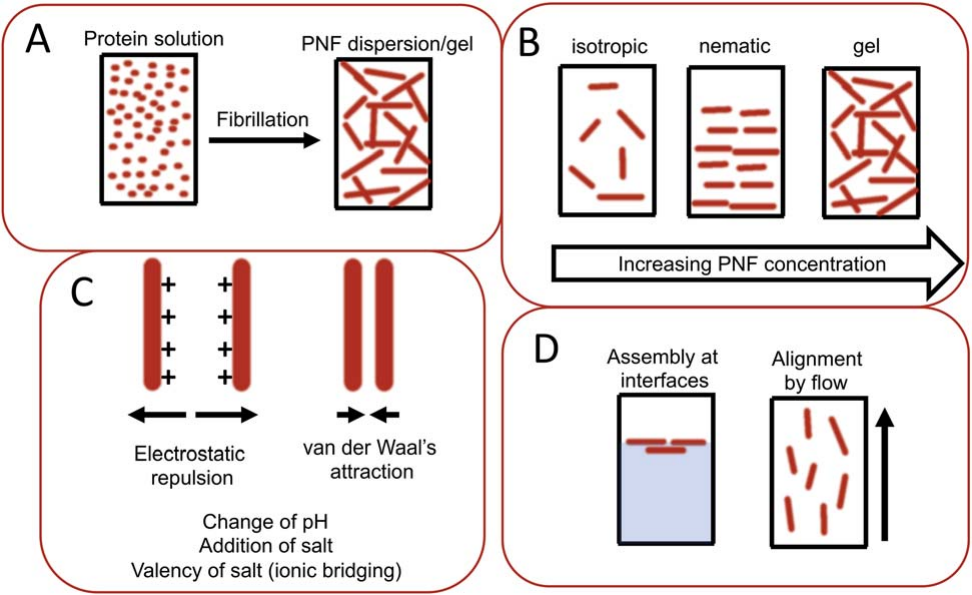
Illustration of important parameters during assembly of PNFs into macroscopic materials. (A) The conversion of a protein into PNF results in changes of physical properties, such as increased viscosity and sometimes even gel-formation. (B) Depending on the PNF concentration different phases can form including non-ordered isotropic phases, liquid crystalline phases such as the nematic phases, or gels. (C) The PNF dispersion is stabilized by electrostatic repulsion, which counteracts close contacts between PNFs where van der Waals interactions will be strong. The electrostatic repulsion can be influence by variations in parameters such as pH, salt concentration, and addition of multivalent ions that may enable ionic bridging. (D) PNFs are amphiphilic and tend to be enriched at interfaces. PNFs may also be aligned by applying flow.

**Fig. 14:**
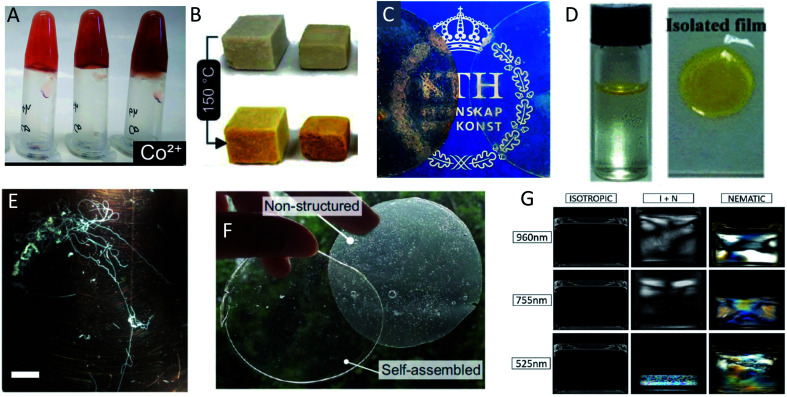
Different examples of macroscopically assembled PNF-materials (A) hydrogels formed from whey fibrillated in the presence of metal ions^129^ adapted with permission from ref. 129. Copyright (2021) American Chemical Society. Further permissions related to the material excerpted should be directed to the American Chemical Society; (B) foams formed by freeze drying of whey PNFs;^216^ (C) free-standing films from whey protein and plasticizer with (left) and without (right) whey-PNFs;^213^ adapted with permission from ref. 213. Copyright (2021) American Chemical Society. (D) An air–water interface film formed from insulin PNFs functionalized with a yellow hydrophobic organic dye. To the right is shown the isolated film^217^ adapted with permission from ref. 217. Copyright (2021) American Chemical Society. Further permissions related to the material excerpted should be directed to the American Chemical Society; (E) microfibers formed by microfluidic wet-spinning of whey PNFs^75^ (scale bar is 1 cm); (F) films formed from soy-protein isolate and plasticizer^123^ adapted from ref. 123. (G) Different phases formed from PNFs of different average length (indicated on the left side) with increasing concentration to the right.^211^ Adpated from ref. 211. Copyright (2018) American Chemical Society.

The assembly of proteins into PNFs results in an aqueous dispersion of fibrils, that can be processed or induced to form a variety of types of macroscopic materials (Fig. 13 and 14). As PNFs are high aspect ratio colloidal particles, they have outstanding potential for fabrication of anisotropic macroscopic materials. In addition, the high aspect ratio promotes inter-fibril contacts already at low PNF concentrations, meaning that PNFs generally have excellent gelation properties, allowing for preparation of hydrogels and aerogels.

Compared to other biopolymers, such as cellulose or silk, PNFs are much less explored as building blocks for engineered materials. The knowledge about how to assemble useful macroscopic materials is still limited – an example of a problematic aspect, which they share with many other engineered protein materials, is that dried PNF-based materials often become brittle. This is in stark contrast to natural protein materials such as silk or wool. However, natural biopolymers such or silk have evolved to provide structural properties that are intimately linked with their hierarchical structural organization. Functional amyloid is also utilized in natural materials, *e.g.* bacteria biofilms^204^ and barnacle cement.^205^ Hence, there is no reason why PNFs should not be able to build up artificial hierarchical materials as well. From that perspective, it is interesting to note recent development where functional amyloid was employed as a part of “aquaplastics”.^206^

There have recently appeared some excellent reviews where the conversion of PNF-dispersions to macroscopic materials are described, in both general and regarding specific aspects,^207^ or specific types of materials such as hydrogels,^208^ and liquid crystals.^209^ However, there are still not many publications focused on formation of (ordered) macroscopic materials from PNFs derived from plants or industrial side-streams. In the present text we will first provide a brief introduction to the assembly of macroscopic materials from PNFs. Then we highlight some examples of PNF-based materials in different formats. When possible we provide examples of PNFs obtained from low cost protein sources such whey protein which apart from being interesting in its own right, can be considered as a model system for “low-purity” protein sources mimicking low-cost protein sources isolated from plants or obtained from industrial side- or waste-streams.

### 6.1 Guidelines for assembly of PNFs into macroscopic materials

The conversion of a protein to PNFs leads to an aqueous dispersion of fibrils (Fig. 13A). The high aspect ratio of PNFs promotes inter-fibrillar contacts that will lead to an increase in the viscosity of the dispersion. At a sufficiently high PNF concentration contacts may form between the fibrils resulting in a continuous network – a gel. PNFs make up an electrostatically stabilized system of high-aspect ratio colloidal particles, that depending on the PNF concentration can form different phases, including isotropic and nematic phases, and gels (Fig. 13B). The net charges of the proteins depend on their amino acid compositions (which define the isoelectric point) and the solution pH but all fibrils will have the same sign of charge. At pH values close to the isoelectric point the net charge of the PNFs will be low, meaning that there will be a reduced stability of the colloidal dispersion and a strong tendency for the PNFs to aggregate. A similar effect can be provided by increase of salt concentration as this will lead to a decrease in electrostatic repulsion. In addition, multivalent ions such as Ca^2+^ and Mg^2+^ can act as intermolecular cross-linking agents, leading to aggregation of PNFs (Fig. 13C).

The anisotropic shape of PNFs provides opportunities to induce formation of ordered structures. High aspect ratio colloidal particles have a strong tendency to form lyotropic liquid crystalline phases, and PNFs are no exception.^209^ The nature of the phase formed is strongly correlate to the PNF aspect ratio, rigidity (persistence length), concentration, and interparticle interactions. High aspect ratio and rigidity promotes formation of a nematic phase. More flexible particles (shorter persistence length) can be approximated by a lower effective aspect ratio. Low PNF concentration favours the isotropic phase and high PNF concentration favours the nematic phase.^210^ PNFs can under certain conditions also form the chiral nematic phase (Fig. 14G)^211^ or grow radially into spherically symmetric particles (spherulites) with micrometer size. External stimuli can be also employed to induce alignment of PNFs. PNFs located in a laminar flow tend to align their long axis parallel to the direction of the flow, and PNFs also have a tendency to become enriched at interfaces between water and a hydrophobic phase, such as air (Fig. 13D).^212^

### 6.2 Macroscopic assembly of PNFs

At a fundamental level, the conversion of the aqueous PNF dispersion into macroscopic material will, with the exception of hydrogels, involve removal of the aqueous phase. As pointed out earlier, materials consisting of tightly packed PNFs tend to be brittle upon drying. One way to avoid this problem is to add low-volatile plasticizers, such as glycerol, that are left behind as water is evaporating. Although this is a well-established strategy in polymer science, limited data is so far available about the plasticization of PNF systems. However, X-ray data from Ye *et al.* indicated that glycerol may alter the structure of the PNFs and “exfoliate” β-sheet structures.^213^ Another way is to employ PNFs as reinforcements in other materials, for example within a synthetic polymer matrix.^214,215^

#### 6.2.1 Formation of hydrogels and aerogels

The earliest examples of PNF materials, are probably found in processing and gelation of food proteins.^218^ Whey-protein isolate and β-lactoglobulin are frequently studied system in this area. β-Lactoglobulin forms particulate and opaque gels when heated at pH close to the isoelectric point, and “fine-stranded” and transparent gels when heated at acidic pH – conditions that are known to promote formation of PNFs.^219^ Interestingly, there are some indications that also the particulate gels have an amyloid-like organization at the molecular level.^220^

The high aspect ratio of PNFs leads to a high probability for formation of inter-fibrillar contacts and under suitable conditions a continuous network of PNFs may form that are surrounded by aqueous medium, resulting in a hydrogel. By removing the aqueous medium, without severely affecting the PNF-network, it is possible to replace water with air, resulting in an aerogel. A standard way of removing water is through freezing followed by freeze drying. The freezing of water leads to the formation of crystal that may deform the PNF-network. The use of critical point drying can reduce this effect; however, deformation of the PNF-network by freezing can also be employed on purpose in order to form structured aerogels, for example by employing directional freezing.^221^

The probability of inter-fibrillar contacts, and thereby the gelling properties, can be controlled by protein (PNF) concentration, temperature, ionic strength, pH and crosslinking additives. The gelation process has been widely studied; for example, Bolder *et al.* and Akkermans *et al.* have studied gel formation as a function of PNF concentration.^121,222^ The effect on gelation of WPI PNFs by additions of divalent cations was studied by Mohammadian *et al.*^223^ Another approach was tested by Ye and co-workers, where WPI was fibrillated in the presence of ions, including multivalent cations (Fig. 14A). The results indicated that the nature of the cation affects the gel properties indirectly by remodeling the assembly of PNFs in the solutions.^129^

An example of formation of hydrogels and their corresponding aerogels from PNFs crosslinked with an organic molecule (multifunctional carboxylic acid that can form amide or ester bonds with the appropriate amino acid residues) is given by Nyström *et al.*^224^ Gels and aerogels from PNFs was prepared by employing CaCO_3_ particles that acted both as physical crosslinks and as a source of Ca^2+^ ions.^225^ Ye and co-workers prepared WPI PNFs, these dispersions were frozen in molds and lyophilized in order to form foams (Fig. 14B).^216^ Interestingly upon aging at 150 °C inter-peptide bonds formed acting as crosslinks giving aerogels high stability towards both temperature and chemicals. This aging process thus helps to overcome problems related to poor mechanical properties and instability of foams towards water. The aged foams are able to retain their properties at 180 °C for as long as 180 h, far exceeding the properties of most classical petroleum-based thermoplastics.

#### 6.2.2 Formation of films

A variety of methods are available for converting colloidal dispersions of fibrillar objects, such as PNFs, into films,^226^ including vacuum filtration, doctor blading, spin coating, and molecular combing where physical forces are employed to promote film formation; or casting from liquid dispersions, followed by evaporation; or by assembly of PNFs at interfaces. In the discussion below we will focus on films robust enough to be isolated (monolayers of PNFs may form at air–water interfaces but these are not strong enough to be isolated).

Vacuum filtration has been developed as a general technique to form films from colloidal systems, and has been employed to form hybrids between PNFs and various carbon-materials. By this method hybrids between PNFs and reduced graphene oxide has been prepared,^227^ as well as hybrids between PNFs and carbon black, resulting in materials that show outstanding efficiency in removal of metal ions (*e.g.* toxic lead ions) from water.^157^

Yuan and Solin demonstrated that PNF films can be deposited on substrates by spray-coating, a method that is easy to scale up. A simple commercial perfume-bottle was employed,^203^ and this methodology will without doubt find increased use employing more sophisticated equipment as employed for example in the case of formation of nanocellulose films.^228,229^

An additional methodology that can enable formation of solid thin films of aligned PNFs is doctor blading, where the PNF dispersion is put between a substrate and a blade. The movement of the blade then leads to a shear flow that will promote PNF alignment in the sample, that upon drying will form a solid film. This approach was employed by Müller and Inganäs to form solid films with aligned hen egg white lysozyme PNF.^230^ The PNFs were functionalized by Congo red, and it was demonstrated by anisotropic absorption of polarized light that Congo red molecules became aligned by the deposited PNFs. Bäcklund and co-workers also utilized doctor blading to prepare films consisting of aligned PNFs functionalized with Nile red, that displayed emission of polarized light.^198^ Due to the alignment of the PNFs, the ensemble of dyes becomes aligned and accordingly the samples exhibit emission of polarized light. In a somewhat related approach it was also possible to employ PDMS-channels to orient insulin PNFs functionalized with 6T.^196^ All these studies demonstrate that thin layers of oriented PNFs can be deposited onto substrates and that the deposited PNFs are able to organize molecular materials (dyes and conjugated polymers) at the nanometer length scale. In addition, Herland and co-workers showed that so called molecular-combing could be employed to form samples of aligned PNFs deposited on substrates. During molecular combing a droplet is deposited onto a substrate and the droplet is then blown off by applying a gas-flow. This will result in a thin coating of PNFs onto the substrate. PNFs could be aligned onto substrates and the PNFs did in turn organize conjugated polymers as demonstrated by emission of polarized light.^231,232^

Spin-coating is a popular procedure to deposit thin polymer films. The methods has for example been used for preparing active layers incorporating PNFs in OLEDs and photovoltaic cells. In contrast to many films formed by other methods, these films are part of a device and the focus is accordingly more on functional performance than the mechanical properties. We are not aware of studies where spin coating is employed to form PNF-only films. The investigated materials systems typically involve relatively polar conjugated polymers that can be dissolved in polar organic solvents (*e.g.* THF or DMSO) that are miscible with water. The polymer dissolved in the co-solvent is then added to the aqueous dispersion of PNFs and upon spin-coating a thin film will form where the PNFs are mixed with the conjugated polymer. In the case of water soluble conjugated polymers a co-solvent is not required.^233^ In a pioneering study, LEDs were prepared where the active layer consisted of an electroluminescent blue emitting polymer and PNFs. Compared to a control device without PNFs the device with PNFs was about 10 times more efficient.^234^ In addition, by employing PNFs functionalized with green- and red-emitting molecules, LEDs capable of emission of white light could be prepared.^235^ Spin coating was also employed to prepare organic photovoltaic cells where PNFs were combined with a conjugated polymer and a fullerene derivative in the active layer.^236^

Free-standing films with nematic ordering were prepared by drop-casting PNFs derived from hen egg white lysozyme or β-lactoglobulin onto polytetrafluoroethylene films.^237^ Films were formed from the PNF dispersion with glycerol or PEG as plasticizers, and when PEG was employed, films with strong nematic order were formed. These films could in turn be functionalized with thioflavin T and the aligned PNFs were able to organize the thioflavin T molecules that as a result displayed emission of polarized light. Another approach was tested by Ye and co-workers where PNFs prepared from whey were combined with a non-fibrillated whey matrix and glycerol as plasticizer (Fig. 14C).^213^ In this manner the influence of the relative volume fractions of PNFs and non-fibrillated protein on physical properties could be studied and it was possible to tune the mechanical properties of the composite. Generally, increasing PNF content leads to a higher Young's modulus and a decreased plasticity. The differences between dialyzed and non-dialyzed PNFs where rather small, indicating that such purification steps might not be necessary for fabrication high performance PNF materials.

Wang and co-workers prepared hybrids between graphene nanoplatelets and PNFs and these could be drop-casted into a mold, and further processed into films that displayed a voltage upon exposure to a temperature gradient, meaning that such materials may be employed as parts of thermoelectric devices.^238^

There are also examples where the protein fibrils are formed simultaneously as the film. A recent contribution from Kamada and co-workers demonstrated that plant derived soy protein isolate (SPI) could be converted into mechanically robust optically transparent metre-scale films (Fig. 14F).^123^ The authors employed a mixed solvent system consisting of 30 v/v% acetic acid, in combination with ultrasonication and heat treatment to overcome the low solubility of SPI. The resulting dispersions could be cast into films that upon cooling gelled and after drying could be isolated as free standing films. To overcome the problem of brittleness glycerol was added. The structural characterization by the authors shows that fibrillar objects are formed, and FTIR spectra display typical characteristics of β-sheet structures. This work illustrates an important challenge in scaling up material production: to reduce the number of processing steps so that nanoscale- and macroscale-structures form at the same time.

Proteins and PNFs are amphiphilic molecules and have a strong tendency to assemble at interfaces. The assembly of proteins at the air–water interface is the basis of many phenomena in cooking.^239^ For example, the preparation of Yuba where soy proteins under heat form thick films at the air–water interface or the protein-skin that may form at the air–water interface when heating milk. Regarding PNFs, several studies have demonstrated that PNFs can form mono-layers at the air–water interface.^212,240^ These tend to be hard to isolate because their mechanical fragility, but nevertheless present an interesting opportunity for formation of extremely thin protein films. In fact, by combining amyloidogenic proteins with gold nanoparticles it was possible to fabricate free-standing monolayers.^241^ In addition, there are new approaches that rely on the *in situ* assembly of amyloid-related structures at the air–water interface to create protein thin films.^242^ Wang and co-workers demonstrated that functionalization of PNFs with hydrophobic dyes promotes formation of films at the air–water interface (Fig. 14D).^217^ The presence of hydrophobic compounds leads to an increased affinity for the air–water interface, and a material that can be characterized as a dense-hydrogel is formed at the air–water interface, with an ordered arrangement of PNFs. The films were sturdy and could be isolated as free-standing films, and the problem of brittleness be avoided by treatment with polyvinyl alcohol (PVA). The ordering of PNFs were retained upon drying. The shape of the container (where the air–water interface assembly occurred) influenced the PNF ordering, and for PNFs functionalized with luminescent dyes, alignment of PNFs resulted in emission of polarized light. It was possible to employ PNFs without purification from hydrolyzed peptide fragments; however, the presence of such fragments led to a lower degree of optical anisotropy in the films.

#### 6.2.3 Formation of fibers

Spinning of micro- or macro-fibers is a common processing method for man made as well as natural materials. Many hierarchically ordered bio-structures in Nature are built up from proteins assembling into fibrils, that in turn assemble into fibers. Typical ways of manufacturing microfibers are through wet-spinning employing microfluidics, or by electrospinning. Such approaches could accordingly lead to artificial protein microfibers that might complement natural silk. In practice the assembly of fibrous materials through wet-spinning has proved challenging (but feasible) also for relatively well-studied classes of materials such as nanocellulose,^243,244^ recombinant spider silk^245^ or a mixture of silk and CNFs.^246^ Kamada and co-workers were able to form microfibers by microfluidic spinning of WPI PNFs, without employing crosslinkers or co-polymers (Fig. 14E).^75^ PNFs were initially dispersed in water at a low pH far from the isoelectric point; by inducing a pH change, approaching the isoelectric point of the protein, a gel transition is induced, that results in the formation of hydrogel-microfibers. Both curved (worm-like) and straight PNFs were employed, and interestingly, only curved PNFs resulted in isolable microfibers. Straight PNFs does form hydrogel-microfibers but these do not cohere when withdrawn from the aqueous media. The curved PNFs may be able to form entanglements that provide higher mechanical resistance to fragmentation. Even when employing curved PNFs, it was necessary to employ purified PNFs with the non-fibrillar peptide fragments removed by dialysis in order to form isolable hydrogel-microfibers.

By employing alginate as a matrix and PNFs from β-lactoglobulin, Kamada *et al.* were later able to produce microfibers from straight fibrils.^247^ Alginate and PNFs were mixed at pH 9, where both components are negatively charged. These materials could then be crosslinked during spinning by addition of Ca^2+^ ions. PNF alignment was influenced by flow-rate, with higher flow-rate giving a higher degree of alignment. The mechanical properties were influenced by the degree of alignment, with a higher degree of alignment leading to a higher Young's modulus.

Electrospinning has also been employed to prepare fibers from a mixture of poly(ethylene oxide) (PEO) and PNFs. It was found that PNFs had a net orientation with the long PNF-axis aligned with the fibers axis, and that incorporation of PNFs gave stiffer fibers.^248^ In another recent study, a coaxial electrospinning approach was used, in which a sheath of polymer solution, with adequate electrospinning properties, encloses the protein core solution. This results in the formation of hybrid fibers with a stabilizing polymer shell and a core made up of PNFs.^249^

As discussed above a wide variety of methods can be employed to form macroscopic PNF materials. The field is still in its infancy and there will no doubt be many additional examples coming employing the various techniques.

## 7. Concluding remarks and future outlook

With this review we hope that we have been able to describe the potential of PNF-based materials. It is indeed fascinating that nanomaterials with sophisticated functional properties can be prepared in processes of similar complexity as cooking a soup. However, the operational simplicity should not be confused with simplicity at the scientific level. The processes involved in PNF formation are highly complex and there are many scientific challenges associated with the conversion of proteins into PNFs. PNFs also combine generic structural properties present regardless of the specific protein employed, with highly specific properties related to the amino acid composition as well as the specific morphology – a highly interesting mix of generality and complexity.

Accordingly PNFs have unique characteristics compared to other biopolymeric materials, and in order to put PNF-based materials into a larger perspective, a short qualitative comparison is here provided between PNFs and two other biopolymers, cellulose nanofibrils (CNF) and DNA. CNFs (and cellulose nanocrystals (CNCs)) are heavily researched for applications both as bulk materials as well as films, fibers and coatings.^250–252^ Such materials, typically generated by top-down deconstruction of wood, may be re-assembled from the bottom-up which may allow for generation of novel structures with novel properties compared to traditional wood-based materials. The high aspect ratio of CNFs, which allows them to be assembled into structurally ordered materials, and their high mechanical strength are attractive properties. However, CNFs are relatively chemically inert, and if chemical reactivity or surface charges are desired, chemical modifications (*e.g.* TEMPO-oxidation) are required. Notably, the first studies on CNFs, where it was demonstrated that cellulose can be deconstructed into a nanomaterials appeared in the early eighties and in the intervening forty years great progress has been made in the reassembly of such nanofibers into advanced materials.^253^ Hence, the timeline for the field of PNF materials is approximately where CNF research were around year 2000.

DNA is another biopolymer that has generated significant interest in materials research.^254–256^ Due to the relatively high cost, the focus is not so much on applications as a bulk-material but rather on relatively high-tech applications such as interface modification in electronic devices or as materials for photonics. These studies are in part motivated by the generation of salmon DNA as an industrial side product. Compared to cellulose nanomaterials, DNA is mechanically weak, and due to the flexibility of the DNA polymer chain it is challenging to assemble DNA into ordered macroscopic materials. On the other hand, DNA contains a range of binding sites where dyes may bind, and DNA can accordingly readily be functionalized with dyes to create materials that might be used for applications in photonics.

Comparing PNFs with DNA and CNFs we find that PNFs seem to combine the most attractive properties of these two materials. Although PNFs are mechanically weaker than CNFs, they are robust structures that because of the high aspect ratio readily forms ordered phases; however, in contrast to CNFs, PNFs are richly endowed with binding sites that can organize *e.g.* dyes at the nanoscale. Hence, PNFs provide a versatile structural platform for various technological applications as bulk materials, but PNFs can also be considered as parts of more complex functional materials or as components of active devices. Hence, PNF-based materials could contribute to sustainability both through replacement of less sustainable bulk materials (*e.g.* as bio-based plastics or novel food) and provide new functionality for sustainable technology (*e.g.* in water purification, photonics, or regenerative medicine). For bulk applications, it will be critical to use raw materials that are available at low cost, in the form of proteins isolated from plants or industrial side-streams. However, an economical value increase can be expected as one goes from applications as bulk materials, to functional materials, to components of active devices. In addition, materials for technological applications will often be applied as hydro/aero-gels, coatings (thin films) or in the form of microfibers, which reduces the material need.

In order to harness the full potential of PNFs it will be important to gain increased structural understanding. With the rapidly increasing number of high-resolution structures, we will obtain improved understanding of how proteins can fold into various structures and what factors that determine the molecular organization in PNFs. It seems that the steric zipper structures formed by shorter peptides are accessible for several different segments within a protein while PNF formation from full length proteins requires close to optimal 2D folding. Increased knowledge in this area will open up for more sophisticated design of PNF structures.

It will moreover be important to gain improved understanding of the relationship between protein molecular structure and the nanoscale morphology of fibrils. This connection is only vaguely understood today. With increased understanding of how a given protein, in combination with fibrillation conditions, influences PNF structure it may be possible to rationally design different types of PNFs. Such knowledge might allow for design of templates that could organize dyes into specific arrangements that may influence the photophysical properties, for example by inducing chirality or formation of specific types of dye aggregates. In addition, for the functional properties (*i.e.* interactions with surroundings) the molecular details of the protein/peptide and the distribution of accessible functional groups become important. In this manner it may be possible to influence the interaction between PNFs and functionalization agents, which may in turn modify *e.g.* the release properties of hybrids between PNFs and drug molecules, nutrients, or pesticides.

Improved methods for assembly of PNFs into macroscopic materials need to be developed. In addition, better understanding is required of how PNFs behaves as polymeric systems on their own or together with other materials in composites, among other things in order to reduce the problem of brittleness. For the design of macroscopic materials, there is no reason to only use one type of protein. Combinations of different types of structures could expand the accessible structure–property space, in analogy with the combination of disordered chains and nanocrystals found in silk. Moreover, little is currently known about the structural integrity of the fibrils in different types of composites or after material processing steps. All of the above points will be important in order to design *e.g.* appropriate mechanical properties of macroscopic samples.

An important part of utilization of protein materials is their attractive points from the perspective of sustainable development. However, it should be noted that, the level of sustainability for a raw material is more complex than just the animal *vs.* plant issue. Several question must be considered, including: (1) what are the actual production conditions in terms of energy/water consumption, animal welfare, working condition of employees? (2) What are the production efficiency and yield? (3) Is it possible to make use of waste streams from existing industries, *e.g.* dairy or vegetable oil production? (4) What are the processing requirements in terms of *e.g.* energy/water consumption? Is it possibilities to use existing production facilities and equipment? (5) Does material production compete with food production (on local or global scale)? (6) Are there potential health risks (in particular for food-related applications)? (7) How can the used product be recycled, and what happens to the waste? Again, more knowledge about the processes is needed to select the best raw materials.

Since essentially any protein can be converted into PNFs, often using simple methodology employing water as solvent, and the resulting PNF dispersion can be processes into various types of macroscopic materials, PNFs will be a valuable addition to the rapidly growing toolbox of “green” methodology for the materials scientist.^257,258^ Such methodology conforms with many of the 12 principles of green chemistry^259^ and presents a convenient route to fabricate “green” nanomaterials with a hierarchical structural order at multiple length scales. However, it is clear that the field needs to advance and acquire better understanding of how we can control the processes to obtain the desired yield, structures and mechanical properties. Such studies also need to move from model systems to potential large-scale proteins resources. With that transition it will also become important to evaluate the influence of side products (*e.g.* small peptide chains from protein hydrolysis) on the performance of PNF-based materials for various applications. As discussed above, different material formats seems to have different sensitivity for non-fibrillar protein residuals. Considering the many times we have referred to recent studies in this review, it is clear that we are right now in a very interesting development phase of this research field.

## Conflicts of interest

There are no conflicts to declare.

## Supplementary Material
